# Site-specific bioalkylation of rapamycin by the RapM 16-*O*-methyltransferase†
†Electronic supplementary information (ESI) available. See DOI: 10.1039/c5sc00164a


**DOI:** 10.1039/c5sc00164a

**Published:** 2015-03-02

**Authors:** Brian J. C. Law, Anna-Winona Struck, Matthew R. Bennett, Barrie Wilkinson, Jason Micklefield

**Affiliations:** a School of Chemistry and Manchester Institute of Biotechnology , The University of Manchester , 131 Princess Street , Manchester , M1 7DN , UK . Email: jason.micklefield@manchester.ac.uk; b Department of Molecular Microbiology , John Innes Centre , Norwich , NR4 7UH , UK; c Isomerase Therapeutics Ltd , Science Village, Chesterford Research Park , Cambridge , CB10 1XL , UK

## Abstract

The methylation of natural products by *S*-adenosyl methionine (AdoMet, also known as SAM)-dependent methyltransferase enzymes is a common tailoring step in many biosynthetic pathways. The introduction of methyl substituents can affect the biological and physicochemical properties of the secondary metabolites produced. Recently it has become apparent that some AdoMet-dependent methyltransferases exhibit promiscuity and will accept AdoMet analogues enabling the transfer of alternative alkyl groups. In this study we have characterised a methyltransferase, RapM, which is involved in the biosynthesis of the potent immunosuppressive agent rapamycin. We have shown that recombinant RapM regioselectively methylates the C16 hydroxyl group of desmethyl rapamycin precursors *in vitro* and is promiscuous in accepting alternative co-factors in addition to AdoMet. A coupled enzyme system was developed, including a mutant human enzyme methionine adenosyl transferase (MAT), along with RapM, which was used to prepare alkylated rapamycin derivatives (rapalogs) with alternative ethyl and allyl ether groups, derived from simple *S*-ethyl or *S*-allyl methionine analogues. There are two other methyltransferases RapI and RapQ which provide methyl substituents of rapamycin. Consequently, using the enzymatic approach described here, it should be possible to generate a diverse array of alkylated rapalogs, with altered properties, that would be difficult to obtain by traditional synthetic approaches.

## Abstract 2

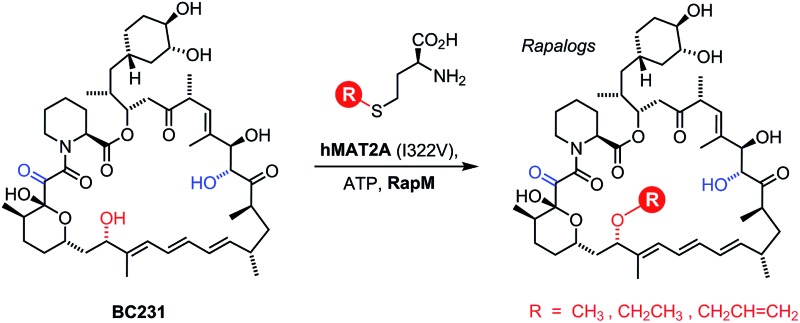
Characterisation of a rapamycin *O*-methyltransferase (RapM) and its utilisation in coupled reactions, with an improved variant of the human methionine adenosyl transferase (hMAT2A), results in new regioselectively alkylated rapamycin derivatives.

## Introduction

The natural product rapamycin **1**, first isolated from *Streptomyces hygroscopicus* NRRL 5491 (reclassified as *Streptomyces rapamycinicus* sp. nov.),^1^,^2^ is a polyketide macrolide antibiotic and a potent immunosuppressant that is widely used in the clinic.^3^,^4^ Rapamycin's mechanism of action derives from its binding to the FKBP12 immunophilin, with the resultant rapamycin-FKBP12 complex acting to inhibit downstream signalling pathways involving the protein kinase mTOR (mammalian target of rapamycin).^5^ Rapamycin analogues (rapalogs) have been approved for the treatment of a variety of diseases, demonstrating the versatility and wide-ranging biological activity of this natural product. For instance, the semi-synthetic derivative Everolimus possesses efficacy against a number of disorders^6^ such as renal angiomyolipoma,^7^ neuroglial and neuroendocrine tumours,^8^,^9^ and breast cancer.^10^,^11^ Other rapalogs possess a shorter half-life for reduced systemic immunosuppression,^12^,^13^ or have displayed neuroprotective abilities and show promising potential as treatment for ischemic stroke victims.^14^ In light of this, there has been considerable interest in the diversification of the rapamycin scaffold to create novel rapalogs with altered or improved physicochemical and pharmacological properties.

Rapamycin is a highly complex molecule and although total syntheses of rapamycin have been reported,^15^,^16^ these syntheses are unlikely to deliver the quantities of rapalogs that would be required for drug development. While semi-synthetic rapalogs have received FDA-approval, the scope and flexibility of this approach is limited with regards to amenable diversification of the rapamycin scaffold. The elucidation of the rapamycin biosynthetic gene functions^17^,^18^ has enabled methods including precursor-directed biosynthesis and mutasynthesis, to deliver rapalogs rapidly and in higher titres by fermentation using engineered strains.^19^–^24^ However, the modifications introduced by these approaches have so far been largely constrained to the cyclohexane starter unit or the pipecolic amino acid, with only a few examples of modifications made to the remainder of the rapamycin scaffold.^18^,^25^

**Fig. 1:**
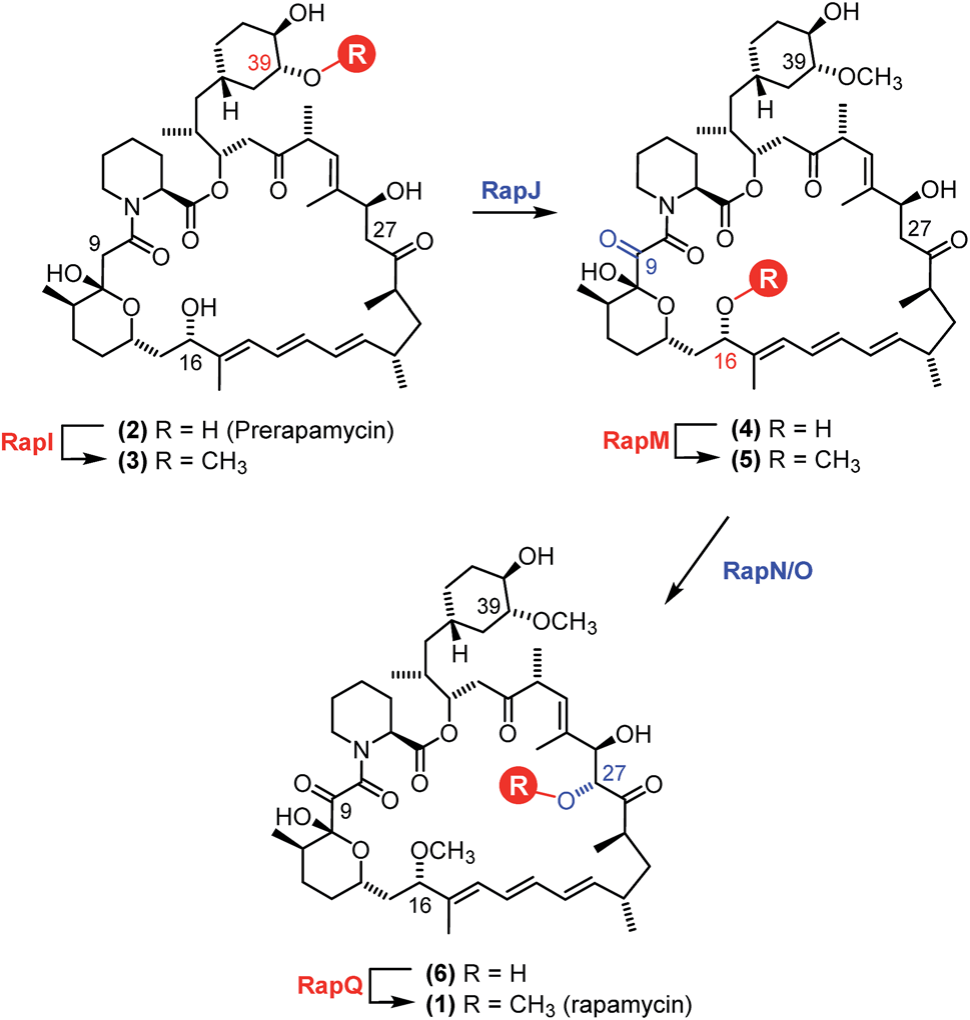
Proposed modification of prerapamycin **2** by tailoring enzymes.^18^ The first post-PKS intermediate **2** is assembled by three PKSs RapA, B and C and cyclised by the pipecolate-incorporating NRPS-like enzyme RapP. Subsequent modification of the macrocycle occurs by methylation at the 16, 27 and 39-*O*-positions by three *O*-methyltransferases RapM, RapQ and RapI. Two P450 monooxygenases RapJ and RapN serve to introduce a keto-group and hydroxyl-group at the C9 and C27 positions respectively. RapQ methylates only after hydroxylation at C27 by RapN.

The polyketide synthase (PKS) enzymes, RapA, B and C along with the nonribosomal peptide synthetase pipecolate incorporating enzyme RapP are responsible for assembly of the macrocyclic intermediate prerapamycin **2**.^17^ Post-PKS tailoring enzymes, including three *O*-methyltransferases RapM, I and Q, and two P450 monooxygenases RapJ and N, are required to transform prerapamycin, the first PKS free intermediate, into the mature macrocycle **1** (Fig. 1).^18^ Gene knockout experiments suggest that RapI, M and Q are regiocomplementary AdoMet-dependent methyltransferases that methylate C39, C16 and C27 hydroxyl groups during the biosynthesis of rapamycin respectively. Given that some AdoMet-dependent methyltransferases have been shown to transfer alternative alkyl groups from AdoMet analogues,^26^–^31^ we envisaged that the regiocomplementary rapamycin methyltransferases might be utilised in the diversification of distal positions of the rapamycin structure.

The natural co-factor AdoMet, utilised by the majority of methyltransferases, is biosynthesised from ATP and l-methionine by the enzyme methionine adenosyl transferase (MAT).^32^ Production of AdoMet analogues *in vivo* carrying alternative alkyl groups on the sulphonium centre has been demonstrated by feeding methionine analogues to engineered *Escherichia coli* (*E. coli*) cells possessing MAT, with the resultant alkyl-donors subsequently utilised by methyltransferases to alkylate proteins.^33^ During the completion of the work described in this manuscript, another study was reported describing the use of MAT enzymes *in vitro*, to generate AdoMet analogues that were used in coupled enzyme reactions with an *O*-methyltransferase that catalysed subsequent alkylations of the C4′-hydroxyl group of the sugar moiety of a biosynthetic desmethyl intermediate of rebeccamycin.^34^ Other reports have utilised synthetic AdoMet analogues as alkyl-donors.^26^–^29^,^35^,^36^ However, the chemoenzymatic route *via* MAT offers several advantages. Firstly, AdoMet analogues produced enzymatically using MAT are diastereomerically pure;^37^ conversely the synthesis of AdoMet analogues, from *S*-adenosyl-homocysteine and alkyl halides, generates a mixture of diastereomers^37^,^38^ at the sulphonium centre, of which only the (*S*)-configured diastereoisomer is accepted by methyltransferases.^37^,^39^ Synthetic AdoMet analogues also need careful purification to remove the unwanted (*R*)-configured diastereoisomer and residual *S*-adenosyl-homocysteine (SAH), both of which can inhibit methyltransferases.^40^,^41^ Additionally, some AdoMet analogues are unstable^29^,^42^,^43^ so an *in situ* enzymatic preparation, using MAT, can minimise the formation of degradation products. Finally, unlike AdoMet, methionine analogues are membrane permeable and can therefore be fed to cells possessing MAT to generate alternative AdoMet derivatives *in vivo*.^33^ Here we describe the first *in vitro* characterisation of a rapamycin *O*-methyltransferase, and its utilisation in coupled reactions with an improved variant of the human enzyme hMAT2A, creating alkylated derivatives of rapamycin at the C16-*O*-position.

## Results and discussion

### Characterisation of the RapM *in vitro*

The wild-type *rapM* gene sequence was amplified from a pUC18-rapM construct (Biotica)^18^ and sub-cloned into the *E. coli* expression vector pET28(a) generating the expression construct pET28a-rapM (Fig. S1A†). The RapM protein was heterologously expressed in *E. coli* BL21 (DE3) cells using standard methods, and the soluble N-terminal His_6_-RapM fusion protein (Fig. S1B†) was subsequently purified using Ni-NTA affinity chromatography.

**Fig. 2:**
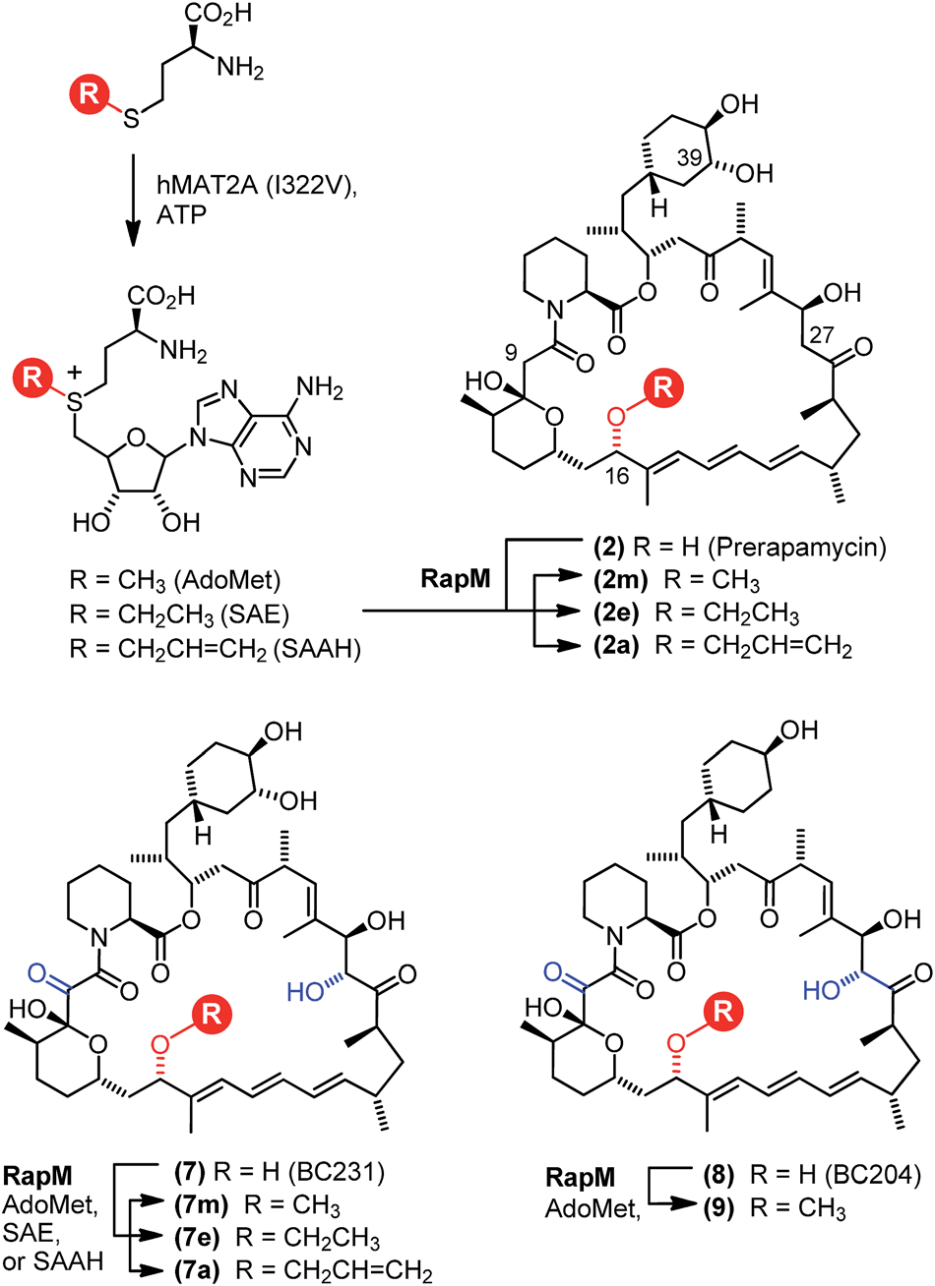
Enzymatic production of AdoMet analogues by MAT and alkylation of rapalogs by RapM. The hMAT2A mutant I322V utilises ATP and methionine analogues to produce AdoMet analogues carrying methyl (AdoMet), ethyl (SAE) and allyl (SAAH) substituents on the sulphonium centre. The AdoMet analogues are subsequently used by RapM to transfer alkyl groups to the rapalog substrates prerapamycin (BC150) **2** and BC231 **7**, generating methyl (**m**), ethyl (**e**) and allyl (**a**) derivatives.

**Fig. 3:**
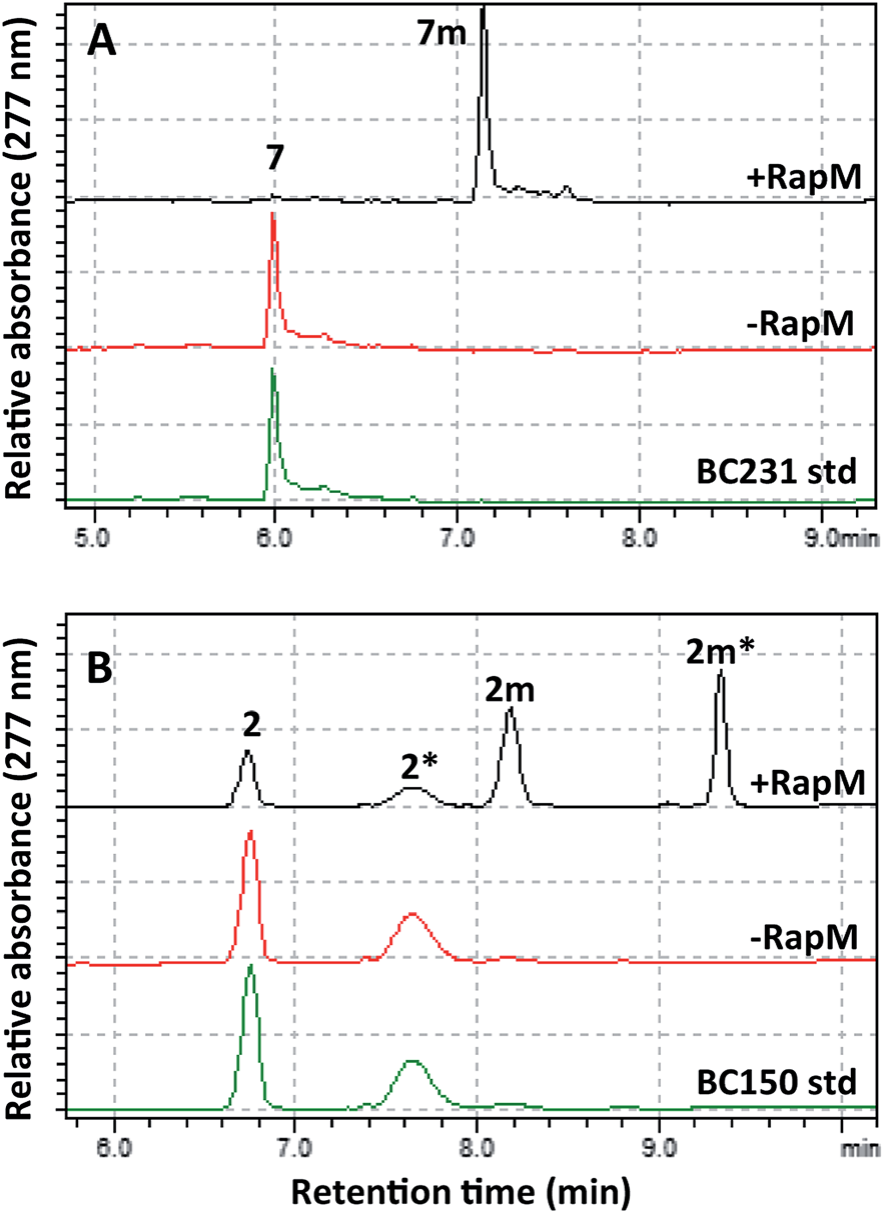
HPLC analyses of BC231 (**7**) and BC150 (**2**) assays with AdoMet. (A) RapM assay with BC231 **7**, showing formation of the methyl product **7m**. (B) RapM assay with BC150 **2** and 2* (rotamers), showing formation of dual peaks corresponding to rotamers of the methylated product (**2m**, 2m*).

The *in vitro* activity of the RapM enzyme was evaluated using AdoMet (Sigma Aldrich UK) as a co-factor and prerapamycin (BC150) **2**, which was previously isolated from an *S. rapamycinicus* deletion strain deficient in the genes encoding the post-PKS tailoring enzymes.^18^ In addition, 16-, 27-, 39-tri-*O*-desmethyl rapamycin (BC231) **7** (Fig. 2), derived from an engineered *S. rapamycinicus* strain lacking the methyltransferase-encoding genes *rapI, M & Q*,^44^ was also evaluated as a substrate for RapM. Analysis of the BC231 **7** reaction by C_8_-reverse phase high performance liquid chromatography (RP-HPLC) (Fig. 3A) revealed formation of a new product with a longer retention time, *t*_R_ = 7.13 min, compared with the starting material *t*_R_ of 5.99 min. The new product was isolated and high resolution mass spectrometry (HRMS) analysis revealed an observed *m*/*z* 908.5156 consistent with the [M + Na]^+^ ion for a mono-methylated derivative of BC231 (Table S1†). Based on the proposed post-PKS tailoring steps (Fig. 1),^18^ this new product was expected to be 16-*O*-methyl-BC231 **7m**. When prerapamycin (BC150) **2** was used as a substrate with RapM, C_18_-RP-HPLC analysis (Fig. 3B) revealed two peaks for the substrate (*t*_R_ = 6.77, *t*_R_ = 7.67 min) due to the presence of interconverting rotamers about the amide bond,^45^ along with new products with longer retention times which were predicted to be rotamers of 16-*O*-methyl-BC150 **2m** (*t*_R_ = 8.22, *t*_R_ = 9.36 min). The two product peaks for **2m** were separated and HRMS revealed the same *m*/*z* for each, consistent with mono-methylated prerapamycin rotamers (Table S1†). Additionally, the individual rotamers of both prerapamycin **2** and product **2m** were separated by HPLC, left at 21 °C for 16 h, and then re-analysed again by HPLC, which revealed re-equilibration back to the original ratio of rotamers (Fig. S2†).

### Confirmation of the regioselectivity of RapM

**Fig. 4:**
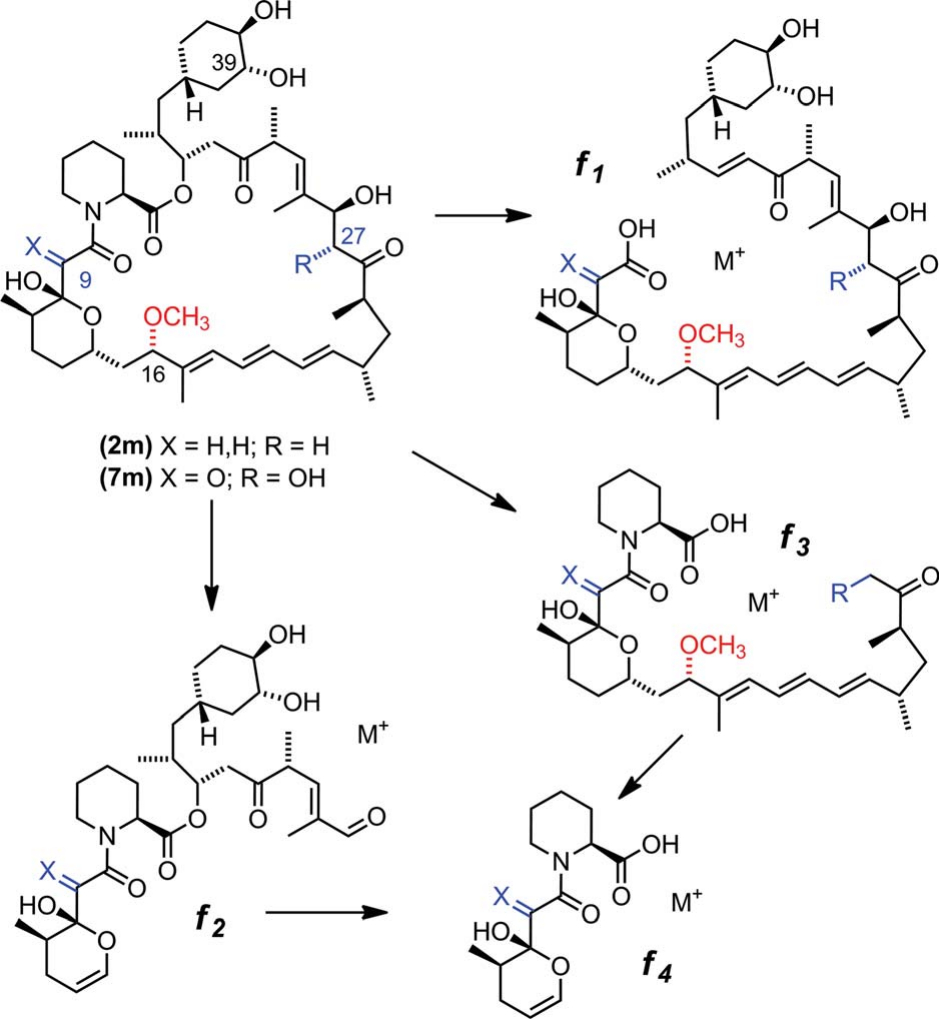
MS^n^ fragmentation pattern for rapalogs, showing the defined product ions f_1_, f_2_, f_3_ and f_4_. The RapM site of methylation can be identified by comparing the f_1_ and f_2_ ions for rapalogs **7**, **7m** and **2**, **2m**.

Based on gene deletion and complementation experiments with *S. rapamycinicus* the three methyltransferases RapM, Q and I, are predicted to methylate at the 16-, 27- and 39-*O*-positions respectively during rapamycin biosynthesis.^18^ However, as of yet there has been no *in vitro* characterisation of these methyltransferases. Therefore, to confirm the regioselectivity of RapM, the mono-methylated products **2m** and **7m** were subjected to tandem mass spectrometry revealing a fragmentation pattern consistent with rapalogs described previously (Fig. 4).^18^ In addition to the key MS/MS product ‘f’ ions of **2**, **2m**, **7** and **7m** (Table 1) a series of ions denoted as ‘e’ ions which result from the loss of CO_2_, H_2_O, and in the case of methylated products CH_3_OH, can also be used to assign structures and identify the methylation site of RapM (Fig. S3 and S4†). The f_2_ product ion containing only the C39-OH site was identical for **2** and **2m** (*m*/*z* [M + Na]^+^ 614.2), as well as both **7** and **7m** (*m*/*z* [M + K]^+^ 644.2), indicating that RapM has not methylated the C39-hydroxyl. The f_1_ and f_3_ ions were both observed with a +14 mass difference between **7** and **7m**, which would be consistent with methylation at either the C16- or C27-hydroxyl groups of compound **7**. However, the MS/MS analyses of **2** and **2m** also revealed a +14 increase in the f_1_ product ion. As prerapamycin **2** lacks the C27-hydroxyl, the presence of an additional methyl group in the f_1_ fragment from **2m** suggests that RapM is indeed the 16-*O*-methyltransferase for rapamycin (Fig. S4†). Furthermore, reactions of RapM with previously isolated^46^ compounds BC204 **8** (16,27-bis-*O*-desmethyl-39-demethoxy-rapamycin) and BC207 **9** (27-*O*-demethyl-39-demethoxy-rapamycin), showed conversion of **8** into **9**, *i.e.* RapM catalyses methylation at the C16-hydroxyl (Fig. 2 and S5A†). The absence of activity of RapM with **9**, a rapalog where the C16-hydroxyl is blocked by methylation but the C27-hydroxyl remains available, further confirms that RapM does not accept the C27-*O*-methylation site and is exclusive for the C16-position (Fig. S5B†).

**Table 1:** MS/MS of rapalogs BC150 (**2**) and BC231 (**7**) alkylation of rapalogs using MAT and RapM in tandem

Ion	Calculated *m*/*z*		Observed *m*/*z*	
	**2** [M + Na]^+^	**2m** [M + Na]^+^	**2** [M + Na]^+^	**2m** [M + Na]^+^
Parent	864.5	878.5	864.4	878.5
f_1_	735.4	749.5	735.3	749.4
f_2_	614.3	614.3	614.2	614.2
f_3_	556.3	570.3	—	—

Ion	Calculated *m*/*z*		Observed *m*/*z*	
	**7** [M + K]^+^	**7m** [M + K]^+^	**7** [M + K]^+^	**7m** [M + K]^+^
Parent	910.5	924.5	910.4	924.4
f_1_	781.4	795.4	781.3	795.3
f_2_	644.3	644.3	644.2	644.2
f_3_	602.3	616.3	602.2	616.2

Finally, the 16-*O*-methyl-BC231 compound **7m** was analysed by proton-NMR (600 MHz), which revealed that **7m** exists as a mixture of amide rotamers, in line with previous NMR studies of rapamycin **1**.^47^ From COSY and TOCSY experiments in DMSO-*d*_6_, most of the ^1^H signals for the major *trans*-isomer of **7m** could be assigned (Table S2 and Fig. S6–S8†) and are consistent with NMR data reported previously for rapamycin **1** as described by Pagano.^47^ Notably a 3H singlet in the ^1^H NMR of **2m** is evident at 3.06 ppm, which corresponds with the C16-*O*-methyl signal assigned previously at 3.04 ppm, for rapamycin in DMSO-*d*_6_. This, along with the MS/MS experiments confirms the regioselectivity of the 16-*O*-methyltransferase RapM.

### Alkylation of rapalogs using MAT and RapM in tandem

To effect regioselective alkylation of rapamycin, a one-pot coupled enzyme system with the human methionine adenosyl transferase hMAT2A and RapM was envisaged. The hMAT2A enzyme has been reported to be promiscuous,^33^,^34^ and further mutagenesis of active site residues around the binding pocket for the AdoMet methyl substituent has been shown to improve activity with methionine analogues possessing larger *S*-alkyl substituents.^33^ Based on this precedent a series of active site mutants of hMAT2A were created by site-directed mutagenesis of a hMAT2A expression vector pNIC28-Bsa4-hMAT2A that was kindly provided by Udo Oppermann (Oxford). Selected mutants were overproduced in *E. coli* Rosetta™ 2, purified by Ni-NTA affinity chromatography, and evaluated with l-ethionine and *S*-allyl-l-homocysteine (Sahc) as substrates, revealing that the I322V mutation provides the highest efficiency with the methionine analogues (Fig. S9†).

**Fig. 5:**
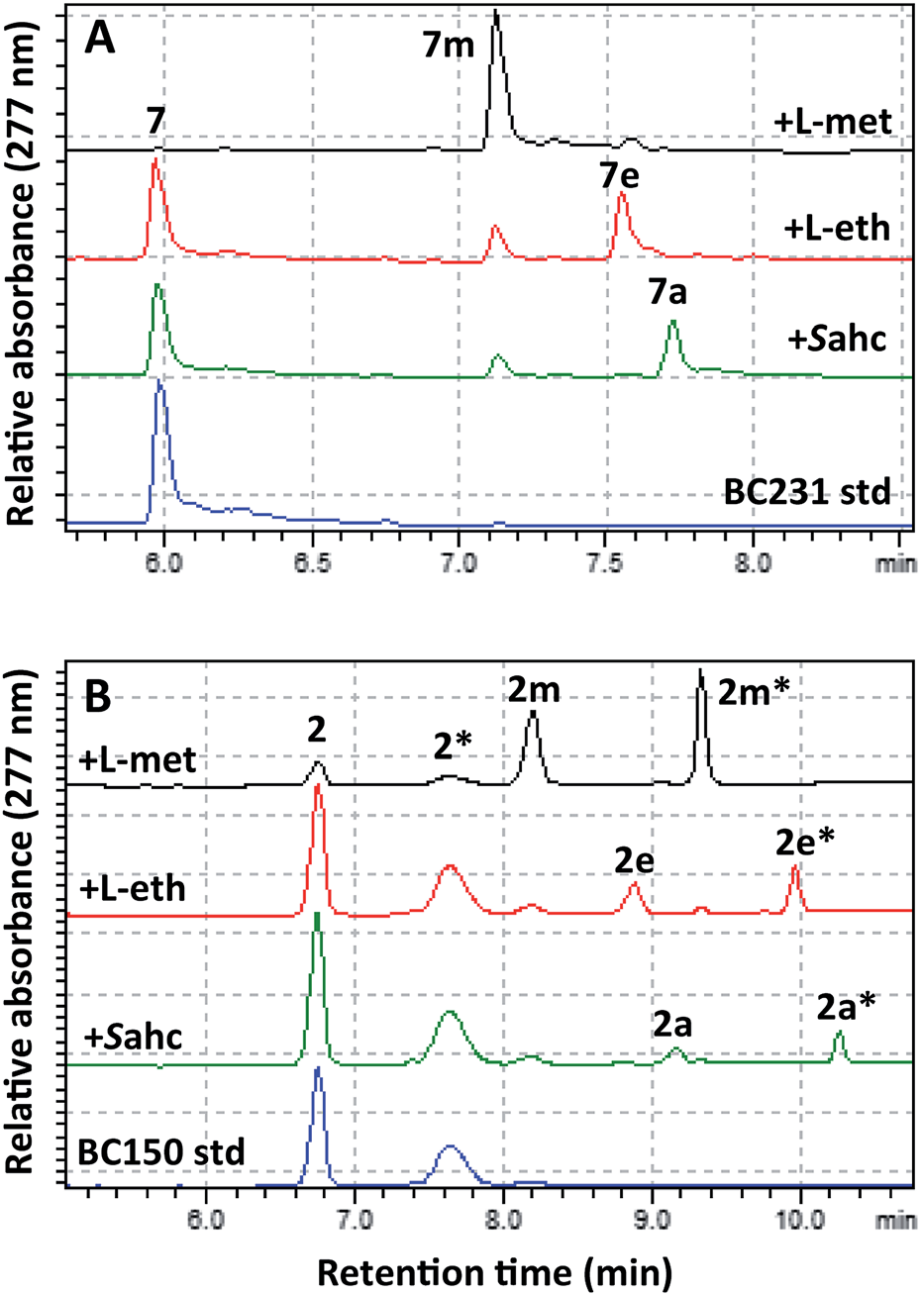
HPLC analyses showing transfer of methyl, ethyl and allyl groups onto the rapalogs BC231 (**7**) and BC150 (**2**). (A) BC231 **7** and methyl **7m**, ethyl **7e**, and allyl **7a** products. (B) BC150 **2** and methyl **2m**, 2m*, ethyl **2e**, 2e*, and allyl **2a**, 2a* products. Peaks labelled with an asterisk denote the secondary rotamer about the amide bond.

The mutant hMAT2A (I322V) and RapM were then used in tandem to effect alkylation of prerapamycin **2** and BC231 **7** with ATP and l-methionine, l-ethionine or *S*-allyl-l-homocysteine, forming *in situ* AdoMet, *S*-adenosyl-l-ethionine (SAE) or *S*-adenosyl-l-allyl-homocysteine (SAAH) respectively. The subsequent transfer of methyl, ethyl and allyl groups onto the rapalogs was analysed by RP-HPLC (Fig. 5). The alkylation of BC231 **7** in coupled assays with hMAT2A (I322V) and methionine analogues resulted in two new products ethyl-BC231 **7e** (*t*_R_ = 7.56 min) and allyl-BC231 **7a** (*t*_R_ = 7.73 min) with longer retention times than the methylated product **7m**. HPLC analysis of the coupled reactions with prerapamycin **2** revealed new products ethyl-BC150 **2e** (*t*_R_ = 8.90 min and 9.99 min) and allyl-BC150 **2a** (*t*_R_ = 9.18 min and 10.28 min). As with the corresponding methylated product **2m**, the ethyl and allyl derivatives also result in dual peaks on HPLC analysis consistent with the presence of rotamers.

The minor amounts of methylated products observed in reactions utilising l-ethionine or *S*-allyl-l-homocysteine are presumably due to the presence of residual active site-bound AdoMet from co-elution during hMAT2A (I322V) and RapM protein purification. HRMS of the new alkylated products **2e**, **2a**, **7e** and **7a** was consistent with mono-alkylated derivatives (Table S1†) and UV analysis showed that all the new products possessed the distinctive rapamycin *λ*_max_ at 267, 277 and 288 nm due to the triene chromophore. Finally partial assignment of the ^1^H and ^13^C NMR signals for the major rotamer of ethyl-BC231 **7e** using COSY, TOCSY, NOESY and HSQC experiments was also consistent with the expected structure of **7e** (Tables S3 and S4 and Fig. S10–S14†). The efficiency of alkylation of the prerapamycin **2** and BC231 **7** in the coupled hMAT2A/RapM assays is clearly reduced with increasing size of the alkyl group on the sulphonium centre of the AdoMet analogue. Full conversion of **7** and 75% conversion of **2** to the methylated rapalogs was achieved for the assays with l-methionine, whereas ethylation and allylation reactions proceed to 19% (**2e**), 51% (**7e**), and 10% (**2a**), 33% (**7a**), under the same conditions (Fig. S15†).

In order to test the efficiency of alkylation with RapM independently of hMAT2A, enzymatically prepared AdoMet, SAE and SAAH (Fig. S16†) were isolated and then separately incubated with RapM and BC231 **7**. Whilst **7m** and **7e** were produced at similar levels to that observed in the tandem reactions, the production of the allylated product **7a** was over five-fold lower compared with the tandem assay (Fig. S17†). To explore this further, competitive assays with AdoMet and equimolar SAE or SAAH were performed (Fig. S18†), which showed that SAE has no effect on the methylation reaction of BC231 **7**. On the other hand, SAAH, and/or the SAAH degradation product 5′-*S*-allyl-5′-thioadenosine^29^,^42^,^43^ (Fig. S16†), appear to inhibit RapM methylation of **7** (Fig. S18†). These observations were used to improve the productivity of the tandem reaction with SAAH (Fig. S19†); by decreasing the concentration of hMAT2A (I322V), with RapM in 7.5-fold excess, yields of **7a** can be increased to 72%. Presumably the excess RapM, relative to hMAT2A, prevents the accumulation of SAAH, minimising degradation and possible subsequent inhibition of RapM.

Finally, the RapM methyltransferase was assayed against a range of concentrations of the best substrate BC231 **7** (2.5–250 µM) with a set concentration of AdoMet (500 µM), to give the kinetic constants *K*_m_ = 48.9 ± 7.1 µM and *k*_cat_ = 1.47 ± 0.072 min^–1^. The relatively low catalytic rate of RapM is unsurprising given the nature of the methylation reaction; RapM is a tailoring enzyme for a complex polyketide natural product, and there is unlikely to be an evolutionary drive for a catalytically efficient methyltransferase, given that the intricate assembly process of the polyketide is more likely to be the limiting factor in overall rapamycin production. The lower relative activity of RapM with AdoMet and the natural precursor BC150 **2** compared with BC231 **7**, an engineered compound possessing a C9 keto group that is not a natural intermediate (Fig. S15†), is also consistent with the proposed timing of tailoring steps in rapamycin biosynthesis.^18^ The proposed optimum pathway (Fig. 1),^18^ suggests RapM methylation occurs after oxidation at C9 by RapJ. Whilst a preferred route may have evolved to ensure each tailoring enzyme acts on its optimal substrate, the fact that BC150 **2** is a substrate for RapM indicates that multiple minor parallel pathways are most likely operative, resulting in a diverse range of intermediary compounds all leading to the final most highly modified product rapamycin.

## Conclusions

In summary, we have characterised the *O*-methyltransferase RapM, confirming its specificity for methylation of the 16-OH position of rapamycin. The ability of RapM to transfer alkyl groups onto substrates including a biosynthetic precursor prerapamycin and an engineered rapalog BC231 has been shown, leading to alkyl-diversification of this clinically important polyketide. In addition, the catalytic constants for the enzyme when assayed with BC231 have been determined with AdoMet.

The enzymatic alkyl-diversification of rapamycin offers an attractive route to generating rapalogs with altered physicochemical and biological activity. Indeed, previous studies^48^,^49^ have noted that structural changes to rapamycin at the C16-position can be well tolerated, and in addition, that groups attached to the C16-hydroxyl group can be localised to a specific region of the rapalog bound FKBP-mTOR complex. Moreover, the regioselective attachment of orthogonal chemical handles to the rapamycin scaffold opens up the possibility of *in vivo* labelling of the rapamycin:FKBP complex, facilitating studies of the interactions of rapamycin with protein targets. Alternatively, the immobilisation of rapamycin offers a new route to coating clinical apparatus such as stents, or allows functional assays of rapamycin in complex with its protein binding partner(s). Given that methyltransferases are one of the most common classes of enzymes found in secondary metabolism, the methods described in this paper could be applied to a wide range of other complex bioactive natural product scaffolds.

## Experimental

### Construction of pET28a-rapM

The wild-type *rapM* gene sequence was PCR amplified using Phusion HF DNA polymerase (New England Biolabs) from a pUC18-rapM construct (Biotica)^18^ with the primers His_6_-rapM *F* and *R* (Table S5†) to introduce *Nde*I and *Xho*I restriction sites at the 5′ and 3′ ends respectively. The gel-purified PCR product was doubly digested with *Nde*I and *Xho*I restriction enzymes (New England Biolabs) and ligated into a linearised pET28a(+) vector (Novagen) which had been similarly digested. The resultant construct pET28a-rapM was verified by nucleotide sequencing (GATC Biotech).

### Site-directed mutagenesis of hMAT2A

The construct pNIC28-Bsa4-hMAT2A (provided by Udo Oppermann, Oxford) was used as a template for site-directed mutagenesis to generate hMAT2A mutants with expanded methionine-binding pockets as described by Wang and co-workers.^33^ Primers carrying the degenerate codon KYT (Table S5†) were designed to generate mutants at the I117, V121 and I322 amino acid positions with either alanine, serine, valine or phenylalanine replacements. The mutants were verified by nucleotide sequencing (GATC Biotech).

### Overexpression and purification of His-tagged RapM and hMAT2A

Transformant cells of *E. coli* BL21 (DE3) (pET28a-rapM) or Rosetta™ 2 (pNIC28-Bsa4-hMAT2A) were cultivated (37 °C, 200 rpm agitation) in LB supplemented with kanamycin (pET28a-rapM, 50 µg mL^–1^) or kanamycin and chloramphenicol (pNIC28-Bsa4-hMAT2A, 50 µg mL^–1^ and 25 µg mL^–1^). The cells were allowed to reach a density of OD_600_ 0.6–0.8 before protein expression was induced with the addition of isopropyl β-d-1-thiogalactopyranoside (IPTG, 0.5 mM). The cells were further cultivated for 4 h at 30 °C, 200 rpm before the cells were harvested by centrifugation. The pelleted cells were resuspended in lysis buffer (5 mL per pellet from 800 mL culture; RapM lysis buffer: 50 mM Tris–HCl pH 8.5, 500 mM NaCl, 5% glycerol (v/v); hMAT2A lysis buffer: 50 mM Tris–HCl pH 8, 50 mM NaCl, 10% glycerol (v/v)). The cells were lysed by sonication and the lysates clarified by centrifugation. The soluble lysate was then applied over a Ni-NTA column (pre-equilibrated in lysis buffer) and washed with lysis buffer containing 20 mM imidazole (7 column volumes, CV) followed with a 60 mM imidazole containing buffer (5 CV). The protein was eluted with 5 CV buffer containing 250 mM imidazole. All fractions were analysed by SDS-PAGE. The eluate fraction containing the RapM or hMAT2A protein was simultaneously buffer exchanged into lysis buffer without imidazole and concentrated using a Vivaspin 20 centricon, MWCO 10 000 (Sartorius Stedim Biotech). The purified enzymes were stored at –80 °C.

### Enzyme activity assays

For activity assays with the commercially purchased co-factor AdoMet (Sigma Aldrich), the RapM methyltransferase was assayed with the rapalogs BC231 **7**, BC150 **2**, BC204 **8** or BC207 **9** as follows: 1 mM DTT, 3 mM MgCl_2_, 1 mM AdoMet, 0.22 mM rapalog, 10 µM RapM, in 20 mM phosphate buffer pH 7.4. The reaction mixes were incubated at 30 °C with 800 rpm agitation, quenched at 30 and 60 min time points with an equal volume of methanol and centrifuged to remove precipitated proteins (13 000 × *g*, 4 °C, 10 min). The reactions were subsequently analysed by C_8_ (rapalogs **7**, **8** and **9**) or C_18_ (rapalog **2**) RP-HPLC.

The hMAT2A assays were run as follows: 1 mM DTT, 3 mM MgCl_2_, 1.5 mM ATP, 1.5 mM l-methionine (or analogue), 10 µM hMAT2A, in 20 mM phosphate buffer pH 7.4. The reactions were incubated at 30 °C, 800 rpm for 30 min, quenched with an equal volume of methanol and clarified by centrifugation (13 000 × *g*, 4 °C, 10 min). The assays were monitored by HILIC HPLC and the AdoMet analogues verified by ES + LC-MS.

For coupled assays of RapM with hMAT2A, the assays were set up accordingly: 1 mM DTT, 3 mM MgCl_2_, 1 mM ATP, 1 mM l-methionine (or analogue), 15 µM hMAT2A (I322V), 0.22 mM rapalog, 15 µM RapM in 20 mM phosphate buffer pH 7.4. The reaction mixes were incubated at 30 °C with 800 rpm shaking, quenched after 30 and 60 min time points with an equal volume of methanol and centrifuged to remove precipitated proteins (13 000 × *g*, 4 °C, 10 min). The reactions were subsequently analysed by C_8_ (rapalogs **7**, **8** and **9**) or C_18_ (rapalog **2**) RP-HPLC.

### NMR of 16-*O*-methyl BC231 and 16-*O*-ethyl BC231

Compounds **7m** and **7e** were prepared by purifying over C_18_ RP-HPLC and collecting fractions containing the product peaks. The fractions were dried under a stream of N_2_ and then *in vacuo*. The dried samples were reconstituted in DMSO-*d*_6_ in a glove bag filled with nitrogen, and transferred to a DMSO-matched Shigemi tube (300 µL sample volume). The NMR spectra were recorded at a temperature of 298 K on a Bruker Avance AMX 600 MHz spectrometer equipped with a 5 mm inverse triple resonance cryoprobe. Spectra processing was performed using Topspin 3.1 software (Bruker).

## Supplementary Material

Supplementary information
