# Supplementary material for: Site-specific bioalkylation of rapamycin by the RapM 16-O-methyltransferase
Source: Chem Sci. 2015 Mar 2;6(5):2885–92. doi: 10.1039/c5sc00164a (PMC5729408; doi:10.1039/c5sc00164a)
Supplement: Supplementary file 1 [file SC-006-C5SC00164A-s001.pdf]

Electronic Supplementary Material (ESI) for Chemical Science.
This journal is © The Royal Society of Chemistry 2015

Site-specific Bioalkylation of Rapamycin by the RapM 16-*O*-Methyltransferase.

Brian J. C. Law,[a] Anna-Winona Struck,[a] Matthew R. Bennett,[a] Barrie Wilkinson,[b,c] and Jason Micklefield[a]*

[a] School of Chemistry and Manchester Institute of Biotechnology, The University of Manchester, 131 Princess Street, Manchester, M1 7DN, United Kingdom

[b] Department of Molecular Microbiology, John Innes Centre, Norwich, NR4 7UH, United Kingdom

[c] Isomerase Therapeutics Ltd, Science Village, Chesterford Research Park, Cambridge, CB10 1XL, United Kingdom

* Email: jason.micklefield@manchester.ac.uk

Supporting Information

Supplementary Methods

All chemicals and reagents were purchased from Sigma Aldrich unless otherwise stated.

HPLC and MS analyses of rapalogs. All enzyme assays were analysed using a Shimadzu Prominence UFLC XR HPLC system unless otherwise stated. The rapalogs BC231 **7**, BC204 **8** and BC207 **9** were analysed using a Shimadzu C_8 Shim-pack XR-ODS column (2.2 µm, 3.0 x 50 mm column, flow rate 1 mL/min, wavelength 277 nm) with the following gradient: 0-8 min (5% B-85% B), 8.1-9 min (95% B), 9.1-10.2 min (5% B), mobile phase A (H_2O + 0.05% TFA), mobile phase B (acetonitrile + 0.05% TFA). The column was maintained at 15 °C. The rapalog BC150 **2** was analysed on a Phenomenex Kinetex XB-C_{18} column (5 µm, 4.6 x 150 mm column, flow rate 0.85 mL/min, wavelength 277 nm) with the following gradient: 0-0.5 min (5% B), 0.5-2 min (5% B-80% B), 2-10.1 min (80% B-95% B), 10.1-11 min (95% B), 11.1-12.2 min (5% B), mobile phase A (H_2O), mobile phase B (methanol). The column was maintained at 25 °C. The rapalog substrates and product peaks were isolated and purified from the assay mixtures by fraction collection using C_{18} RP-HPLC: Agilent Eclipse plus C_{18} column (3.5 µm, 4.6 x 100 mm column, flow rate 1 mL/min, wavelength 277 nm) on an Agilent Infinity 1200 LC system with the following method: 0-3 min (5% B-80% B), 3.1-10 min (80% B-

95% B), 10.1-12 min (5% B), mobile phase A (H_2O), mobile phase B (methanol) and analysed by ES+ MS using a Bruker amaZon ETD. Samples were injected by direct infusion using a Hamilton syringe pump. For high resolution MS, the samples were run on a Waters micromass LCT (ES+).

HPLC and MS analyses of AdoMet analogues. The AdoMet analogues generated by the hMAT2A assays were analysed using a Phenomenex Luna HILIC column (5 µm, 4.6 x 150 mm column, flow rate 1 mL/min, wavelength 260 nm) with the following gradient: 0-1 min (10% B), 1-3 min (10% B-35% B), 3-8 min (35% B-60% B), 8-10 min (60% B), 10.2-11.6 min (10% B), mobile phase A (acetonitrile), mobile phase B (ammonium formate 5 mM, pH 3.3). The AdoMet analogues were verified by LC-MS, using an Agilent 1100 LC-MSD SL system with an Agilent Eclipse plus C_{18} column (3.5 µm, 4.6 x 100 mm column, flow rate 1 mL/min, wavelength 260 nm) and the following gradient: 0-6 min (5% B-25% B), 6-7 min (25% B-95% B), 7 -9 min (95% B), 9.1-11 min (5% B), mobile phase A (H_2O + 0.1% formic acid), mobile phase B (acetonitrile + 0.1% formic acid). High resolution LC-MS was carried out using a Waters Acquity UPLC system (column: Waters C_{18} BEH 1.7 µm, 2.1 x 100 mm, flow rate 0.3 µL/min) with the following gradient: 0-6 min (5% B-70% B), 6-7 min (70% B-95% B), 7 -8 min (95% B), 8-9 min (95% B-5% B), 9-10 min (5% B) mobile phase A (H_2O + 0.1% formic acid), mobile phase B (acetonitrile + 0.1% formic acid). The MS was a Thermo Scientific LTQ Orbitrap XL.

Synthesis, purification and characterisation of the methionine analogue *S*-allyl-L-homocysteine.[43] L-homocystine (268 mg, 1 mmol) was dissolved in liquid ammonia (30 mL) at -78 °C in a dry ice/acetone bath. Sodium metal (100 mg) was added until a dark blue colour persisted for 15 minutes. Ammonium chloride was added (20 mg) until the dark blue colour of the mixture faded. Allyl bromide (0.26 g, 186 µL, 2.15 mmol) was added and the mixture was stirred at -78 °C for 2 hrs. The cooling bath was removed, in order to allow NH_3 to evaporate. The resulting white solid was dissolved in water (30 mL) and extracted with diethyl ether (2 x 10 mL). The pH of the aqueous phase was adjusted to pH 6 with 4 M HCl and 1 M NaOH and the volume was reduced under reduced pressure. The solution was left for crystallisation to give the title compound (120 mg, 0.685 mmol, 45 %) as a white solid. M. Pt: 243-245 °C (lit:[33] 244-245 °C); δH (400 MHz, D_2O/NaOD) 5.62-5.51 (1 H, m, H-6), 4.95- 4.90 (2 H, m, H-7, H-7'), 3.97 (1 H, t, J 6.3 Hz, H-2), 2.97 (2 H, d, J 7.3 Hz, H-5, H-5'), 2.42 (2 H, t, J 7.7 Hz, H-4, H-4'), 2.05-1.90 (2 H, m, H-3, H-3'); δC (100 MHz, D_2O) 171.38 (C- 1), 133.64 (C-6), 117.27 (C-7), 51.6 (C-2), 33.21 (C-5), 29.17 (C-3), 24.90 (C-4); V_{max}/cm^{-1}; 3297.14 (N-H), 2871.22 (C-H), 1667.19 (C=C), 1522.19, 1372.71, 1178.56, 1023.78, 941.23, 845.63, 796.41, 786.69, 612.31, 546.79; *m*/*z* (ES+) 176 $[M+H]^+$, *m*/*z* (ES-) 174 $[M-H]^-$ ($C_7H_{12}NO_2S$ requires 174.24).

RapM assays with pre-prepared AdoMet analogues. Comparison of RapM activity with the AdoMet and analogues, in the absence of hMAT2A, was carried out. Accordingly, AdoMet, SAE and SAAH were prepared using the hMAT2A (I322V) enzyme (15 μM), which was incubated with 1 mM L-methionine, L-ethionine or *S*-allyl-L-homocysteine (Sahc) in phosphate buffer (pH 7.4) containing 1 mM DTT, 3 mM $MgCl_2$, 1 mM ATP, for 60 min at 30 °C, 800 rpm. The assays were transferred into Amicon® Ultra-0.5 centrifugal filter devices (3,000 MWCO) and centrifuged at 14,000 *x g*, 30 min to remove the enzyme. The flowthroughs containing AdoMet, SAE or SAAH were immediately injected onto HILIC-HPLC and quantified against an AdoMet calibration curve (Figure S16). The AdoMet analogues were adjusted to the same starting concentration as AdoMet, with phosphate buffer containing DTT and $MgCl_2$, and RapM (15 μM) and BC231 **7** (0.2 mM) were added to initiate individual alkyltransferase reactions. The assays were run and terminated as previously described. Whilst the conversions of **7** to **7m** and **7e** were similar to those observed with the tandem assays, the formation of **7a** was over five-fold lower when using pre-prepared SAAH compared with the *in situ* formation of SAAH in tandem assays (Figure S17).

Competitive assays involving co-incubation of AdoMet with SAE or SAAH. The enzymatically prepared AdoMet analogues SAE and SAAH, prepared as described above, were separately spiked with an equimolar amount of AdoMet prior to addition of RapM and BC231 **7**. A control assay was set up with AdoMet alone with RapM and BC231 **7**, and the competitive assays were run as previously described. The reactions were monitored by RP-HPLC, showing that SAE does not affect the RapM methylation of **7**, but the presence of SAAH reduces the conversion to **7m** by three-fold (Figure S18). The AdoMet analogue SAAH was found to degrade more readily than AdoMet or SAE, and LC-HRMS of an SAAH sample indeed showed significant formation of a 5′-*S*-allyl-5′-thioadenosine (ATA) degradation product consistent with previous studies[29,42,43] (Figure S16). Thus, it suggested that either SAAH, or its degradation product ATA, inhibit the RapM catalysed methylation of **7**, at higher concentrations.

Optimisation of tandem hMAT2A/RapM allylation of BC231 (7). In light of the observed inhibitory effects of SAAH/ATA on the RapM catalysed methylation of **7**, tandem assays were set up to minimise the amount of 'free' SAAH formed during the allylation of **7** . The assays were set up as described previously, except hMAT2A (I322V) was used at either 2 μM or 15 μM concentration, with 15 μM RapM. The formation of **7a** was monitored by RP-HPLC at 0, 30, 60, 120, 210 and 1110 min time points. The assays containing 2 μM hMAT2A reached 72% conversion of **7** to **7a** at the final time point, whereas the assays containing 15 μM hMAT2A reached 52% under the same conditions (Figure S19). Presumably, the excess of RapM relative to hMAT2A ensures that any SAAH formed is turned

over by the methyltransferase, preventing build-up of SAAH and degradation to ATA which could potentially inhibit RapM activity.

Supplementary Figures and Tables

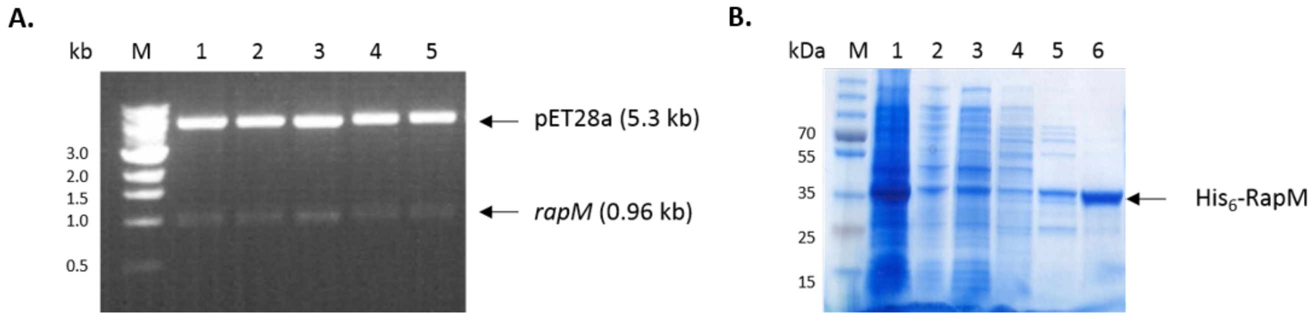

Figure S1. A: *Nde*I and *Xho*I restriction double digest of pET28a-rapM showing presence of the *rapM* insert; **B:** SDS PAGE gel of purified His$_6$-RapM soluble recombinant protein (37.3 kDa).

Table S1. HRMS of rapalogs.

Rapalogue	Ion	Formula	Calculated *m/z*	Observed *m/z*
**7** BC231	$[M+Na]^+$	$C_{48}H_{73}NNaO_{13}^+$	894.4974	894.4955
**7m** Methyl-BC231	$[M+Na]^+$	$C_{49}H_{75}NNaO_{13}^+$	908.5131	908.5156
**7e** Ethyl-BC231	$[M+Na]^+$	$C_{50}H_{77}NNaO_{13}^+$	922.5287	922.5298
**7a** Allyl-BC231	$[M+Na]^+$	$C_{51}H_{77}NNaO_{13}^+$	934.5287	934.5317
**2** BC150	$[M+Na]^+$	$C_{48}H_{75}NNaO_{11}^+$	864.5232	864.5233
**2*** BC150	$[M+Na]^+$	$C_{48}H_{75}NNaO_{11}^+$	864.5232	864.5239
**2m** Methyl-BC150	$[M+Na]^+$	$C_{49}H_{77}NNaO_{11}^+$	878.5389	878.5408
**2m*** Methyl-BC150	$[M+Na]^+$	$C_{49}H_{77}NNaO_{11}^+$	878.5389	878.5366
**2e** Ethyl-BC150	$[M+Na]^+$	$C_{50}H_{79}NNaO_{11}^+$	892.5545	892.5523
**2e*** Ethyl-BC150	$[M+Na]^+$	$C_{50}H_{79}NNaO_{11}^+$	892.5545	892.5568
**2a** Allyl-BC150	$[M+Na]^+$	$C_{51}H_{79}NNaO_{11}^+$	904.5545	904.5513
**2a*** Allyl-BC150	$[M+Na]^+$	$C_{51}H_{79}NNaO_{11}^+$	904.5545	904.5486

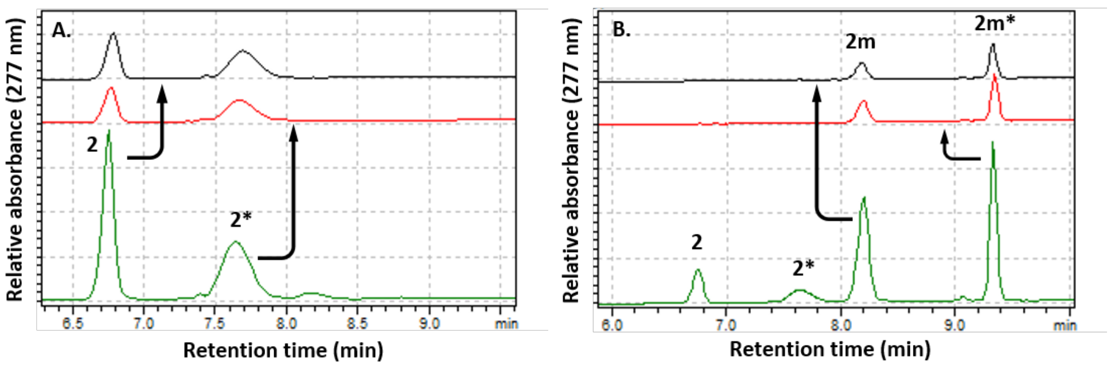

Figure S2. Separation of the rotamers of **A:** BC150 (2, 2*) and **B:** 16-*O*-methyl-BC150 (2m, 2m*) by RP-HPLC. Each rotamer peak was isolated and left at room temperature (21 °C) for 16 h before analysing by HPLC. The chromatograms show that each individual peak re-equilibrates to the original ratio of rotamers when left in solution.

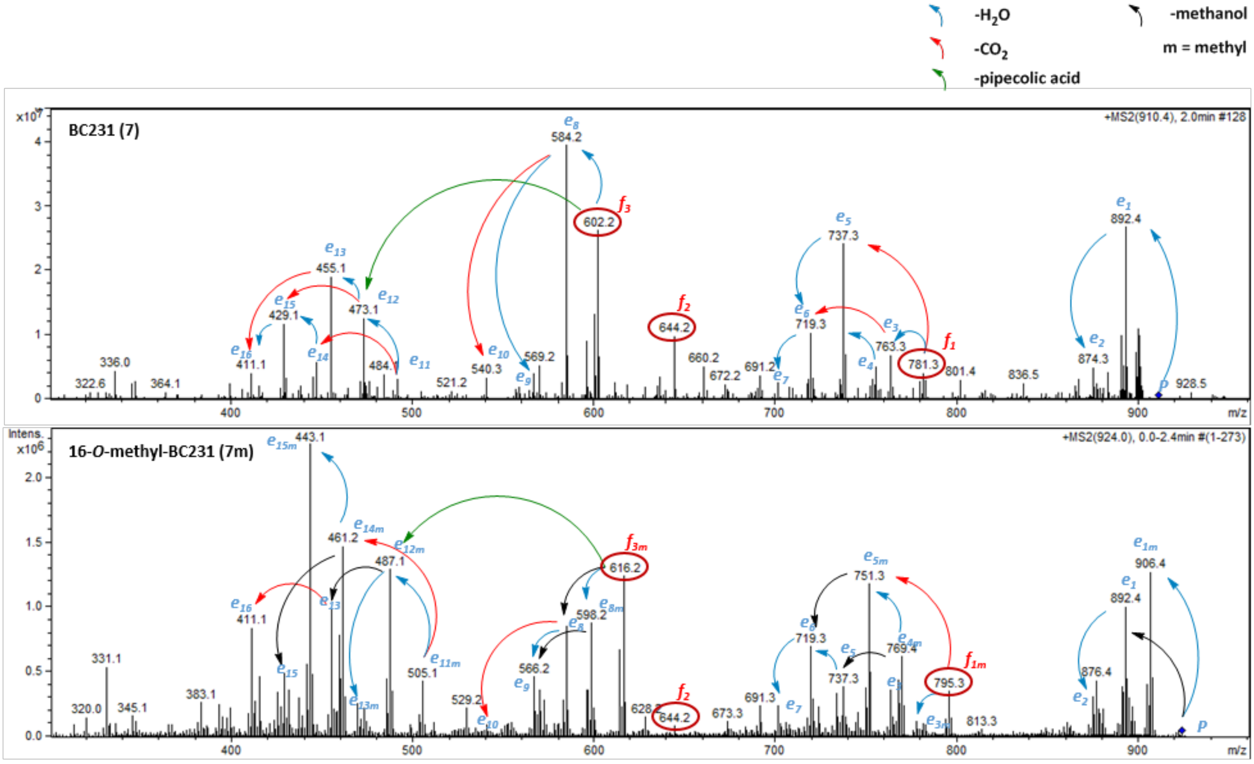

Figure S3. MS^2 analysis of the $[M+K]^+$ ions of BC231 (**7**) and 16-*O*-methyl-BC231 (**7m**). The $[M+K]^+$ ions (*P*) for **7** and **7m** were selected for fragmentation. The identities of fragments f_1, f_2 and f_3 are as previously described, with the **7m** ions showing +14 where methylation is present on the 16-*O*-position. Ions that appear in both the **7** and **7m** spectra with the same *m/z* are highlighted in blue and have been assigned as '*e*' ions and form a fragmentation profile for rapamycin. The 'e_m' ions found only in the **7m** spectrum reflect a mass increase in the fragment ions containing the 16-*O*-position due to methylation. Losses of H_2O, CO_2, CH_3OH and pipecolic acid are denoted by arrows (see key). Comparison of fragment ion clusters between **7** and **7m** show additional losses of CH_3OH where methylation is present in the **7m** sample.

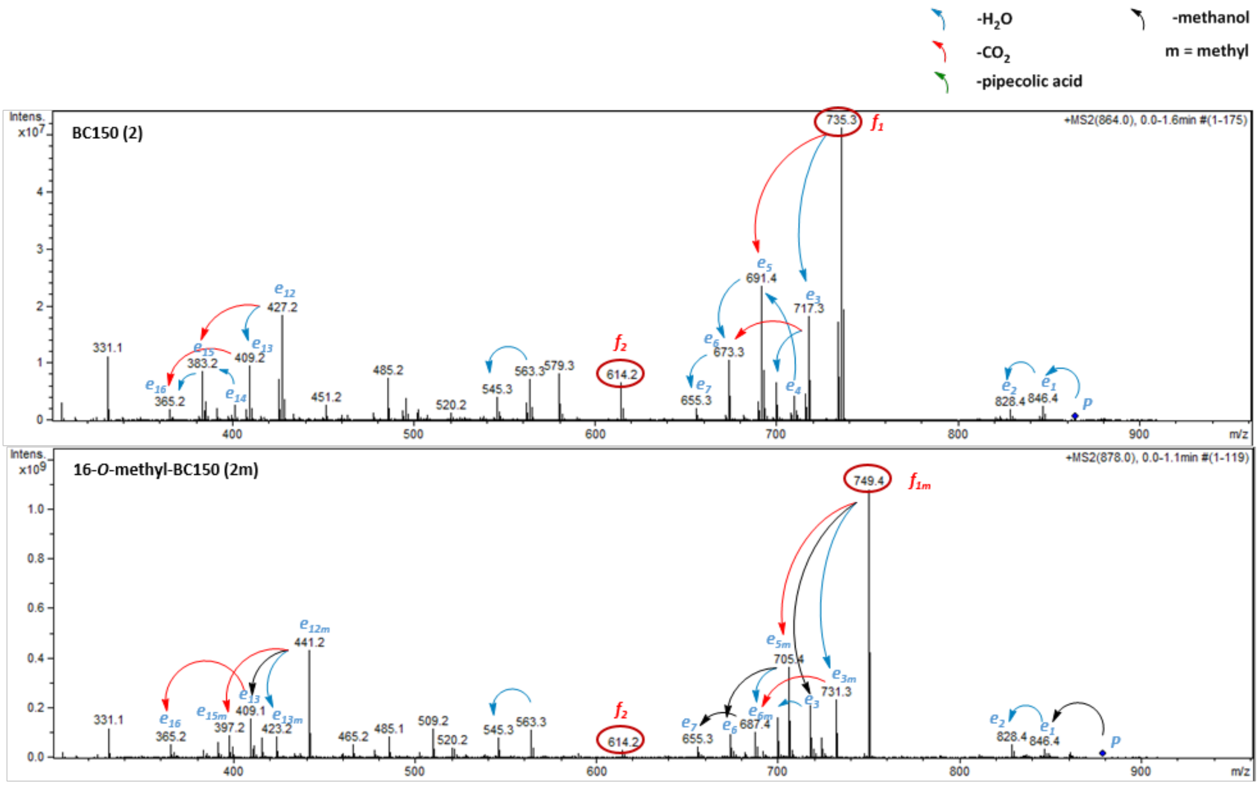

Figure S4. MS^2 analysis of the $[M+Na]^+$ ions of BC150 (**2**) and 16-*O*-methyl-BC150 (**2m**). The $[M+Na]^+$ ions (*P*) for **2** and **2m** were selected for fragmentation. The identities of fragments f_1 and f_2 are as previously described, with the **2m** ions showing +14 where methylation is present on the 16-*O*-position. Ions that appear in both the **2** and **2m** spectra with the same *m*/*z* are highlighted in blue and have been assigned as '*e*' ions and form a fragmentation profile for rapamycin. The 'e_m' ions found only in the **2m** spectrum reflect a mass increase in the fragment ions containing the 16-*O*-position due to methylation. Losses of H_2O, CO_2, CH_3OH and pipecolic acid are denoted by arrows (see key). Comparison of fragment ion clusters between **2** and **2m** show additional losses of CH_3OH where methylation is present in the **2m** sample.

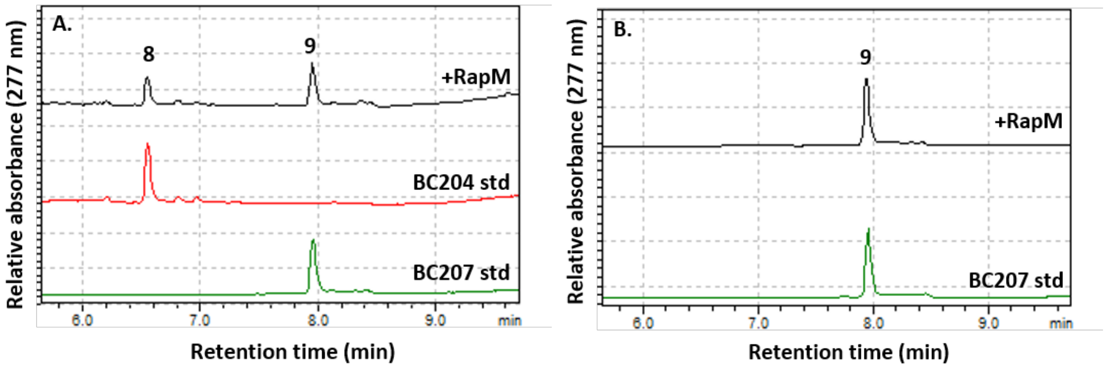

Figure S5. RapM activity with rapalogs BC204 (**8**) and BC207 (**9**). **A:** RapM assay with AdoMet and BC204 **8** possessing C16-OH and C27-OH, showing conversion to **9**; **B:** RapM assay with AdoMet and BC207 **9** possessing C16-OCH_3 and C27-OH, showing no conversion of substrate.

Table S2. Spectral δ ^{1}H partial assignments for 16-*O*-methyl-BC231 (7m) compared with rapamycin (1). The carbon numbering for (7m) and (1) are shown below. The δ ^{1}H assignments for 1 (in DMSO-d_6) are as described by Pagano.[47]

7m

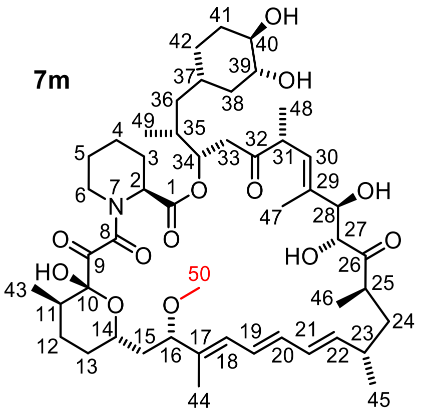

1

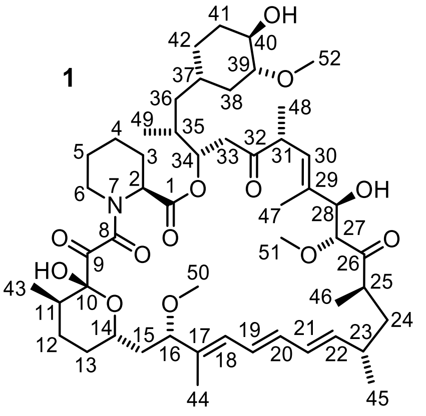

C	δ^1H methyl-BC231 (7m)	δ^1H rapamycin (1)	C	δ^1H methyl-BC231 (7m)	δ^1H rapamycin (1)
1	-	-	28	-	3.99
2	-	4.92	28-OH	-	5.23
3	-	2.08, 1.56	29	-	-
4	-	1.65, 1.38	30	5.10	5.08
5	-	1.55, 1.26	31	3.24	3.27
6	-	3.41, 3.15	32	-	-
7	-	-	33	-	2.72, 2.37
8	-	-	34	4.95	4.97
9	-	-	35	1.64	1.66
10	-	-	36	1.26, 0.99	1.03, 0.94
10-OH	-	6.43	37	-	1.23
11	2.02	2.01	38	-	1.88, 0.58
12	1.52	1.51, 0.83	39	-	2.81
13	-	1.80, 1.16	39-OH	-	-
14	-	3.99	40	-	3.16
15	-	1.83, 1.24	40-OH	-	-
16	-	3.61	41	-	1.73, 1.15
17	-	-	42	-	1.52
18	-	6.10	43	0.75	0.72
19	6.24	6.39	44	1.64	1.62
20	6.40	6.21	45	0.98	0.97
21	6.12	6.12	46	0.84	0.82
22	5.45	5.45	47	1.78	1.73
23	2.25	2.20	48	0.88	0.86
24		1.39, 1.02	49	0.78	0.77
25	2.46	2.39	50	3.06	3.04
26	-	-	51	-	3.15
27	-	3.92	52	-	3.31
27-OH	-	-			

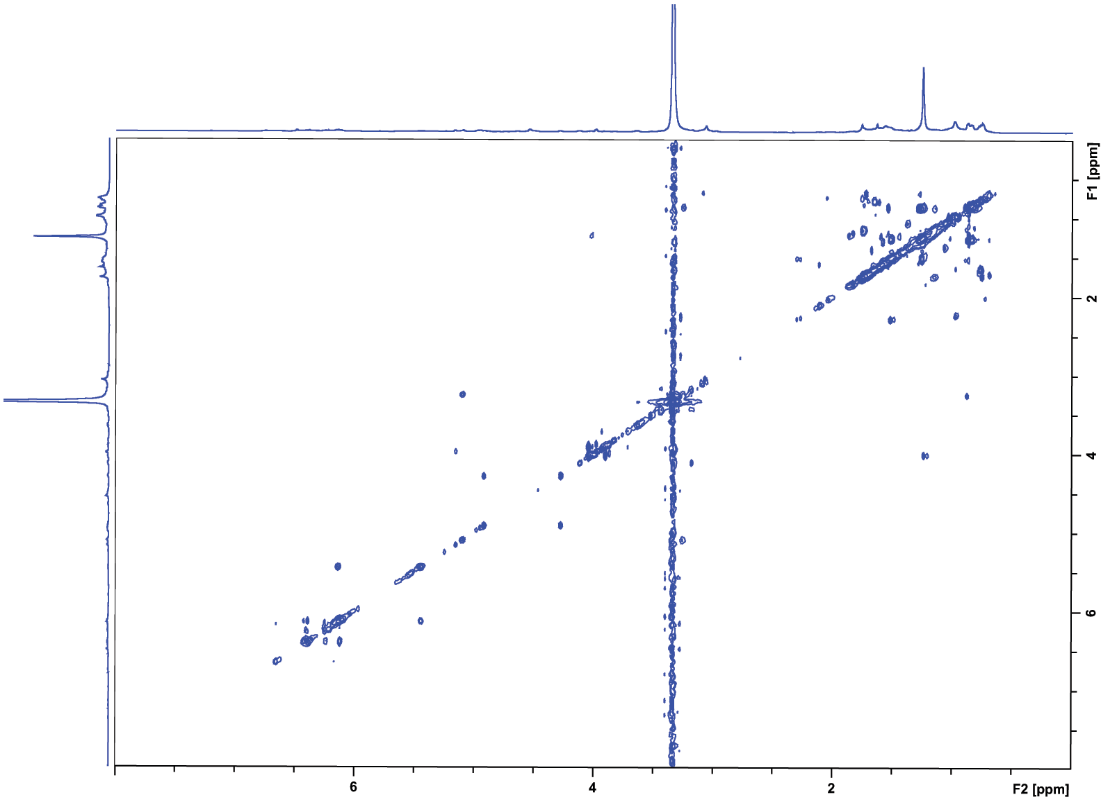

Figure S6. H,H COSY NMR of 16-*O*-methyl-BC231 (**7m**) with Watergate solvent-suppression of the DMSO-d_6 signal. The spectrum was recorded with a total number of scans NS = 16 and with TD(F2) = 4096 and TD(F1) = 512.

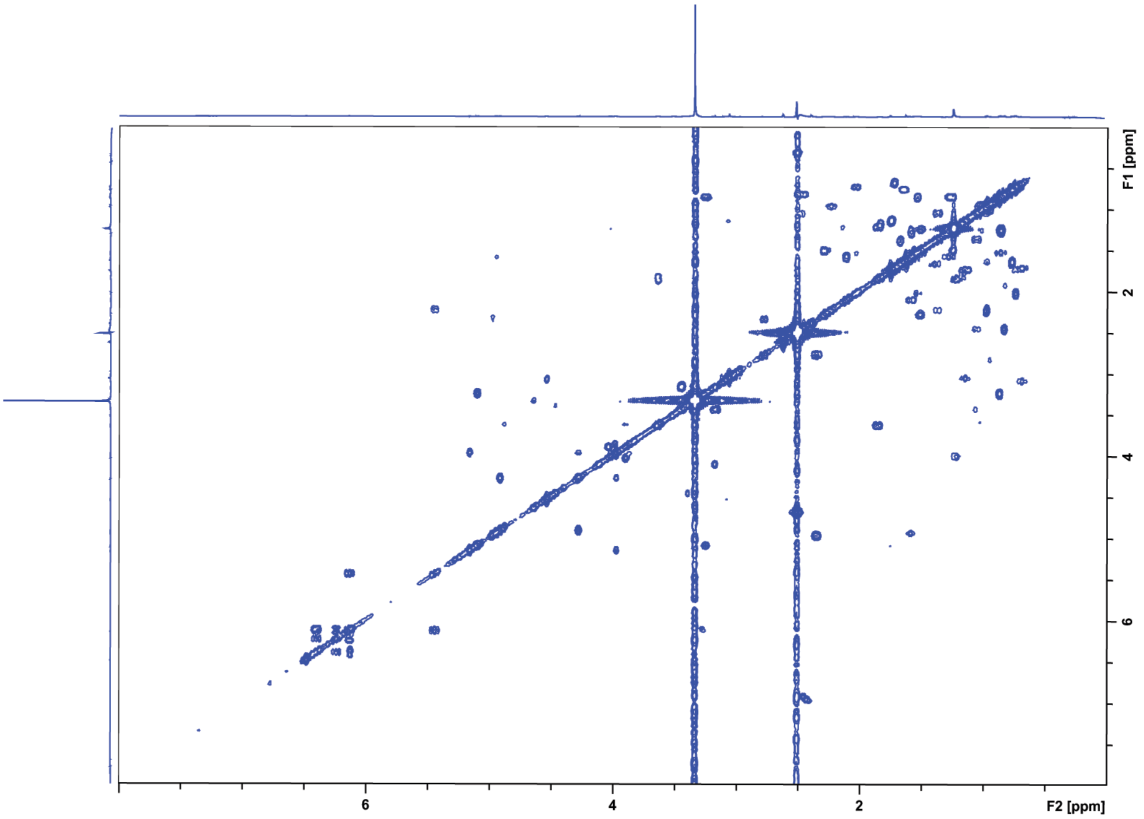

Figure S7. H,H COSY NMR of 16-*O*-methyl-BC231 (**7m**) with preset-suppression of the DMSO-d_6 signal. The spectrum was recorded with a total number of scans NS = 16 and with TD(F2) = 2048 and TD(F1) = 512.

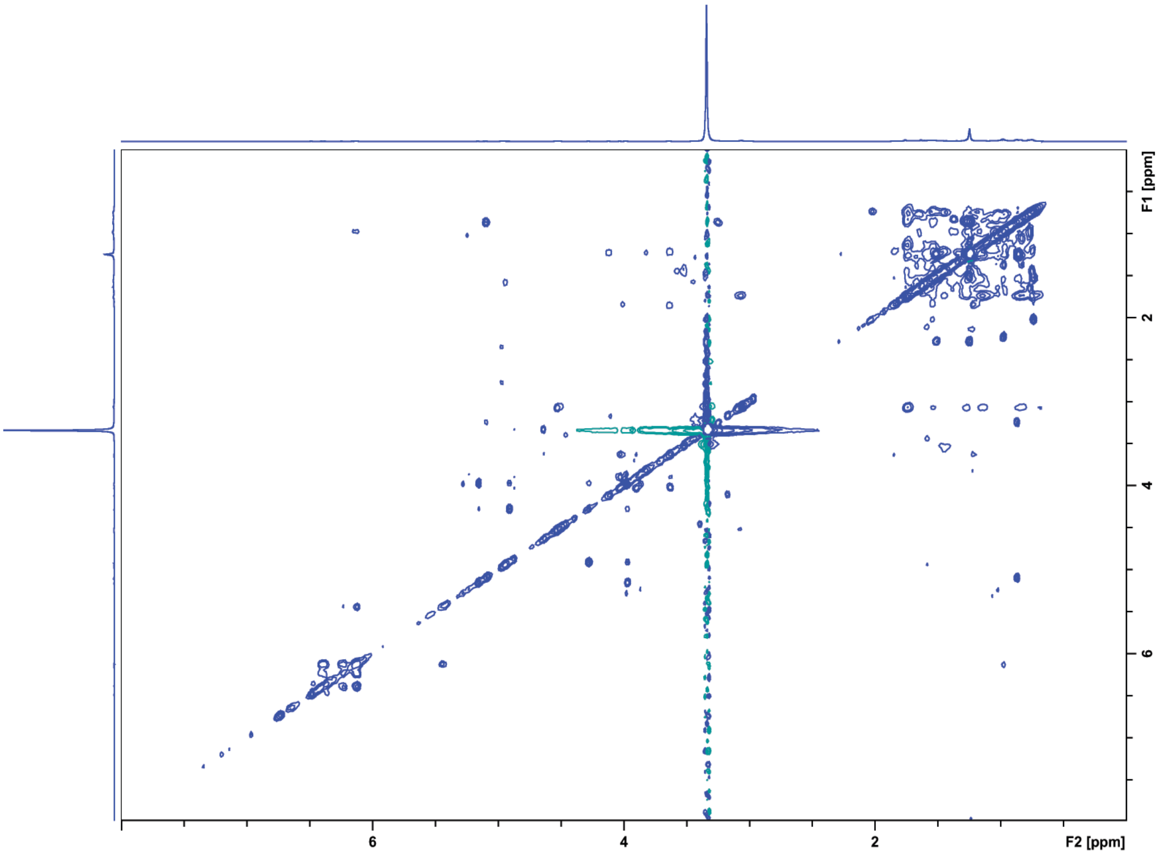

Figure S8. H,H TOCSY NMR of 16-*O*-methyl-BC231 (**7m**) with Watergate solvent-suppression of the DMSO-d_6 signal. The spectrum was recorded with a total number of scans NS = 16 and with TD(F2) = 4096 and TD(F1) = 512.

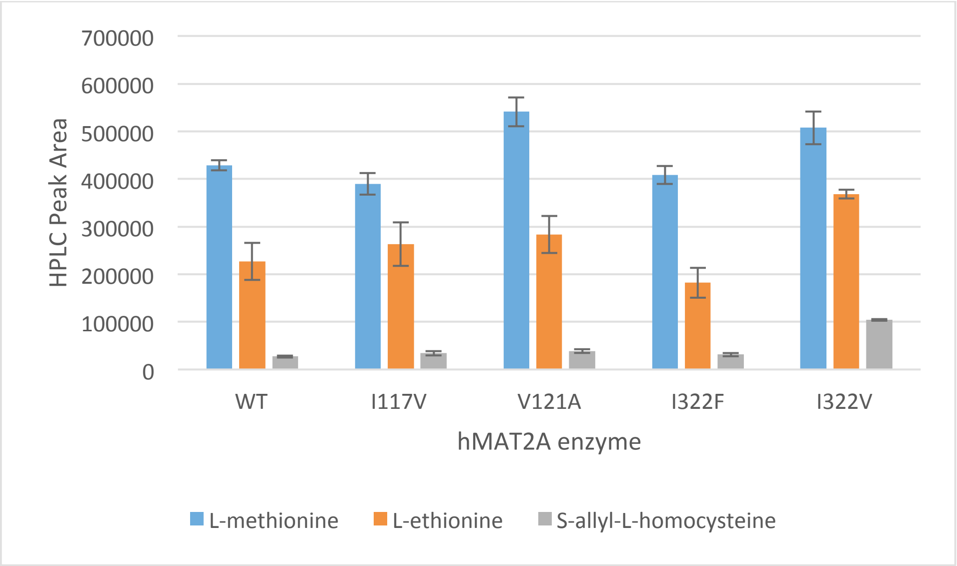

Figure S9. Comparison of hMAT2A wild-type and mutant enzyme activity. The enzymes were assayed with L-methionine, L-ethionine and *S*-allyl-L-homocysteine, and analysed by HILIC-HPLC under identical conditions. The integrated HPLC peak areas for AdoMet, SAE and SAAH were compared between wild-type and mutant hMAT2A as a measure of relative activity. The I322V mutant was selected for RapM/MAT tandem assays due to its greater acceptance of methionine analogues compared with the wild-type and other mutants.

Table S3. Spectral δ^1H partial assignments for 16-*O*-ethyl-BC231 (7e) compared with rapamycin (1). The carbon numbering for (7e) and (1) are shown below. The δ^1H assignments for 1 (in DMSO-d_6) are as described by Pagano.[47]

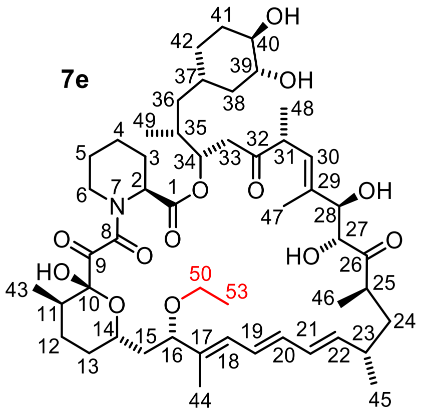

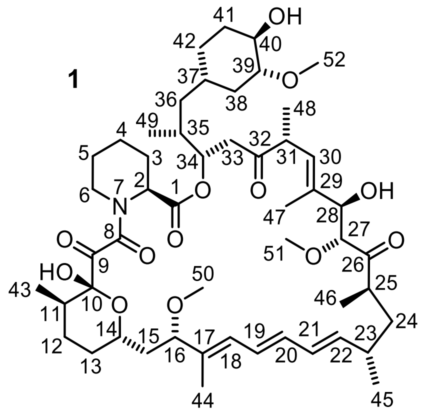

C	δ^1H ethyl-BC231 (7e)	δ^1H rapamycin (1)	C	δ^1H ethyl-BC231 (7e)	δ^1H rapamycin (1)
1	-	-	28	4.11	3.99
2	-	4.92	28-OH	-	5.23
3	-	2.08, 1.56	29	-	-
4	-	1.65, 1.38	30	5.25	5.08
5	-	1.55, 1.26	31	3.57	3.27
6	-	3.41, 3.15	32	-	-
7	-	-	33	-	2.72, 2.37
8	-	-	34	-	4.97
9	-	-	35	-	1.66
10	-	-	36	0.82	1.03, 0.94
10-OH	-	6.43	37	1.28	1.23
11	1.75	2.01	38	0.81	1.88, 0.58
12	1.46, 1.24	1.51, 0.83	39	-	2.81
13	1.25	1.80, 1.16	39-OH	-	-
14	3.64	3.99	40	-	3.16
15	1.38	1.83, 1.24	40-OH		-
16	3.65	3.61	41	-	1.73, 1.15
17	no	-	42	1.15, 1.73	1.52
18	6.17	6.10	43	0.76	0.72
19	6.47	6.39	44	1.57	1.62
20	6.36	6.21	45	0.99	0.97
21	6.14	6.12	46	0.92	0.82
22	5.52	5.45	47	1.62	1.73
23	2.37	2.20	48	0.97	0.86
24	1.54, 1.65	1.39, 1.02	49	0.78	0.77
25	2.82	2.39	50	3.15	3.04
26	no	-	51		3.15
27	3.17	3.92	52	-	3.31
27-OH	-	-	53	1.03	-

Table S4. Spectral δC assignments for 16-*O*-ethyl-BC231 (7e) compared with rapamycin (1). The carbon numbering for (7e) and (1) are shown below. The δC assignments for 1 are as described by Pagano.[47]

C	δC ethyl-BC231 (7e)	δC rapamycin (1)	C	δC ethyl-BC231 (7e)	δC rapamycin (1)
1	-	169.18	28	-	75.72
2	-	50.74	28-OH	-	-
3	-	26.41	29	-	137.12
4	-	20.35	30	-	124.93
5	-	24.45	31	-	45.19
6	-	43.48	32	-	207.52
7	-		33	-	39.94
8	-	166.97	34	-	73.56
9	-	198.87	35	-	33.35
10	-	98.99	36	40.33	38.39
10-OH	-		37	33.67	32.51
11	35.44	34.78	38	31.88	35.43
12	27.32	26.22	39	-	83.73
13	29.16	29.61	39-OH	-	-
14	66.33	66.19	40	-	73.21
15	40.26	40.13	40-OH	-	-
16	80.82	82.24	41	-	32.88
17	-	137.84	42	32.9	31.09
18	-	126.97	43	16.48	15.52
19	-	127.02	44	11.28	10.45
20	128.36	132.30	45	20.05	21.63
21	127.11	130.41	46	16.24	13.39
22	-	139.28	47	15	13.39
23	33.93	35.18	48	16.94	15.56
24	21.72	39.62	49	15.7	14.71
25	-	39.57	50	-	55.44
26	-	210.49	51	-	56.92
27	-	85.52	52	-	56.73
27-OH	-	-	53	15.7	-

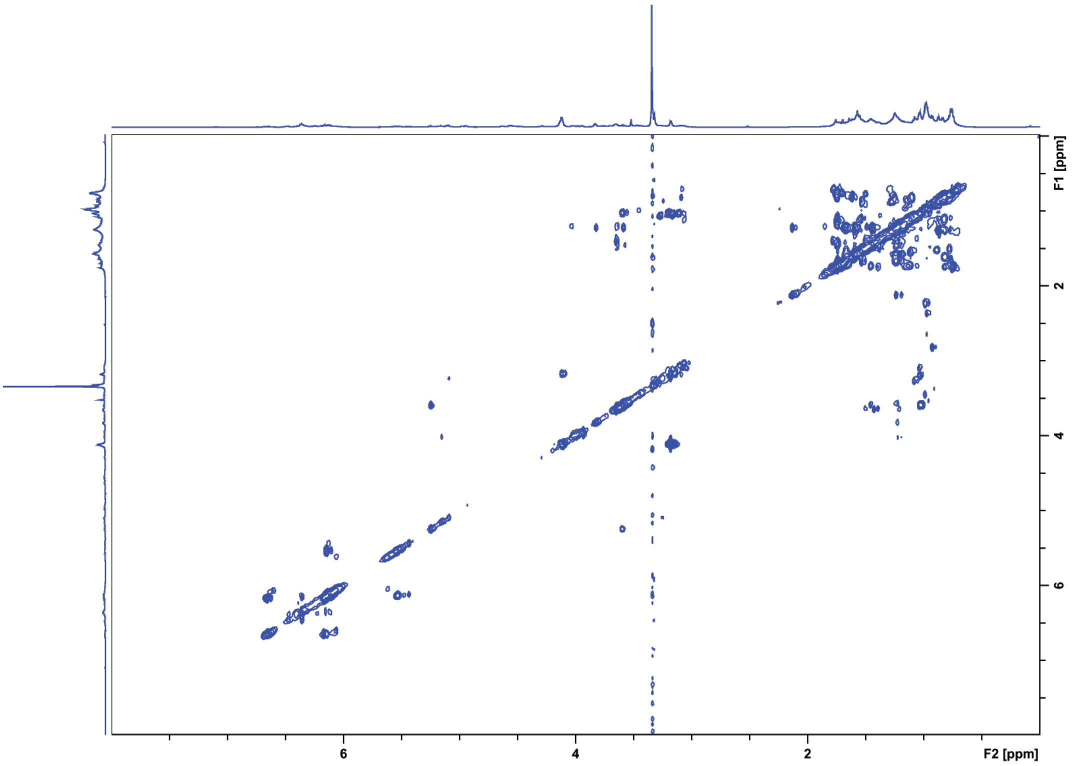

Figure S10. H,H COSY NMR of 16-*O*-ethyl-BC231 (**7e**) with Watergate solvent-suppression of the DMSO-d_6 signal. The spectrum was recorded with a total number of scans NS = 16 and with TD(F2) = 2048 and TD(F1) = 512.

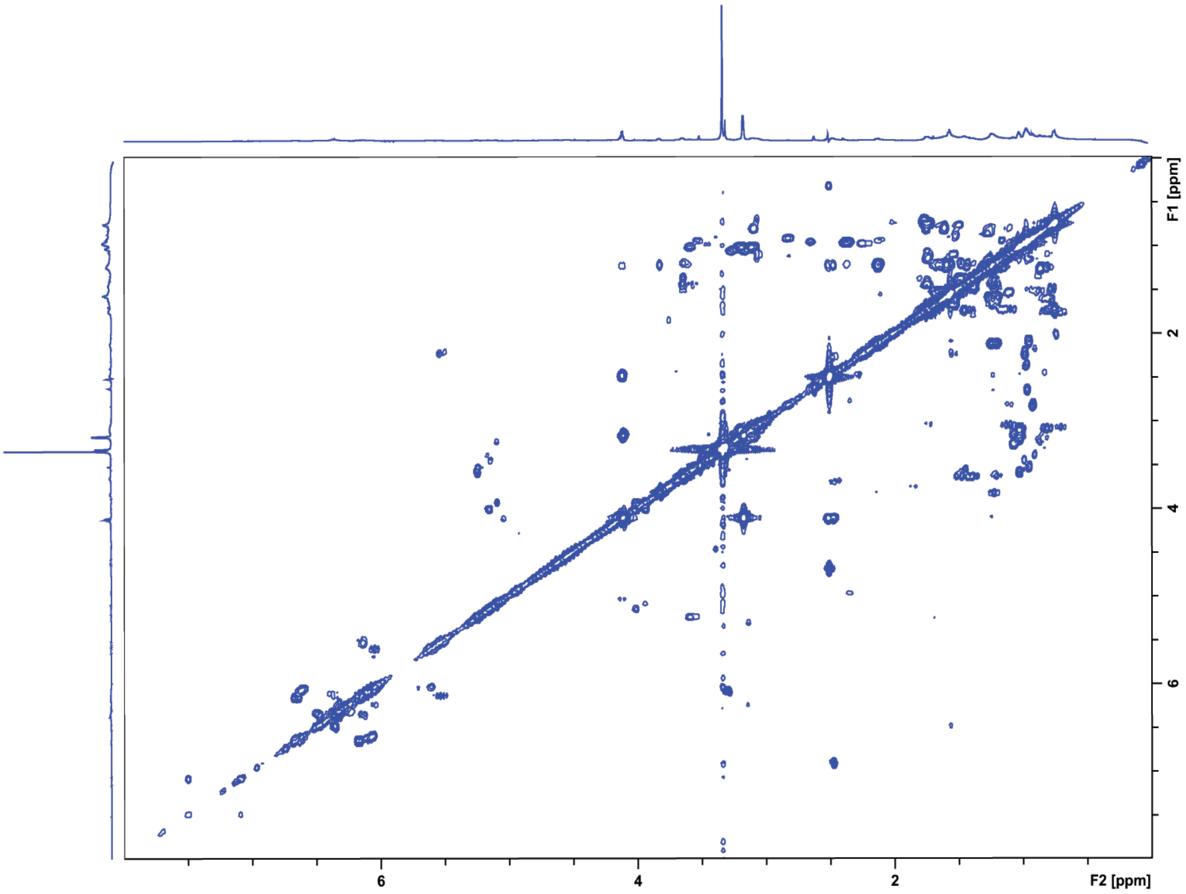

Figure S11. H,H COSY NMR of 16-*O*-ethyl-BC231 (**7e**) with preset-suppression of the DMSO-d_6 signal.

The spectrum was recorded with a total number of scans NS = 16 and with TD(F2) = 2048 and TD(F1) = 512.

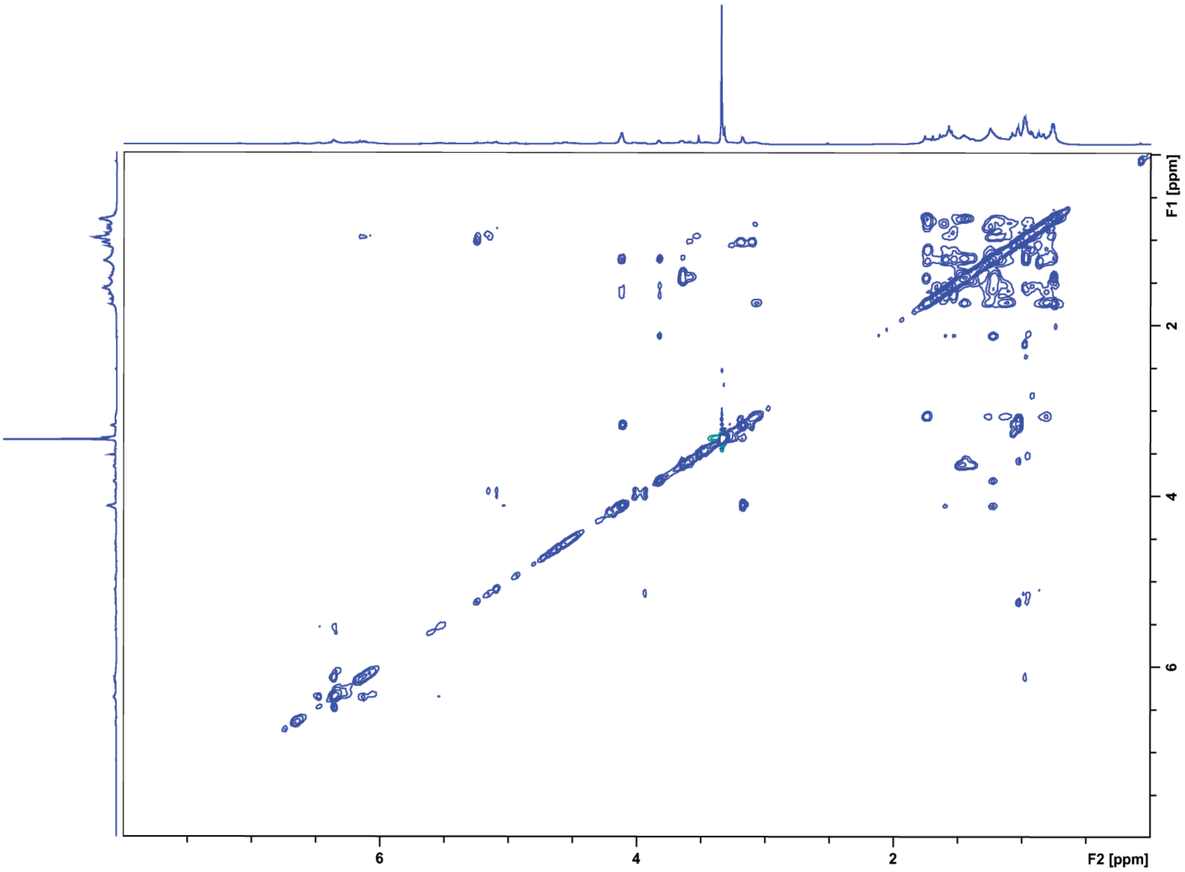

Figure S12. H,H TOCSY NMR of 16-*O*-ethyl-BC231 (**7e**) with Watergate solvent-suppression of the DMSO-d_6 signal. The spectrum was recorded with a total number of scans NS = 16 and with TD(F2) = 2048 and TD(F1) = 512.

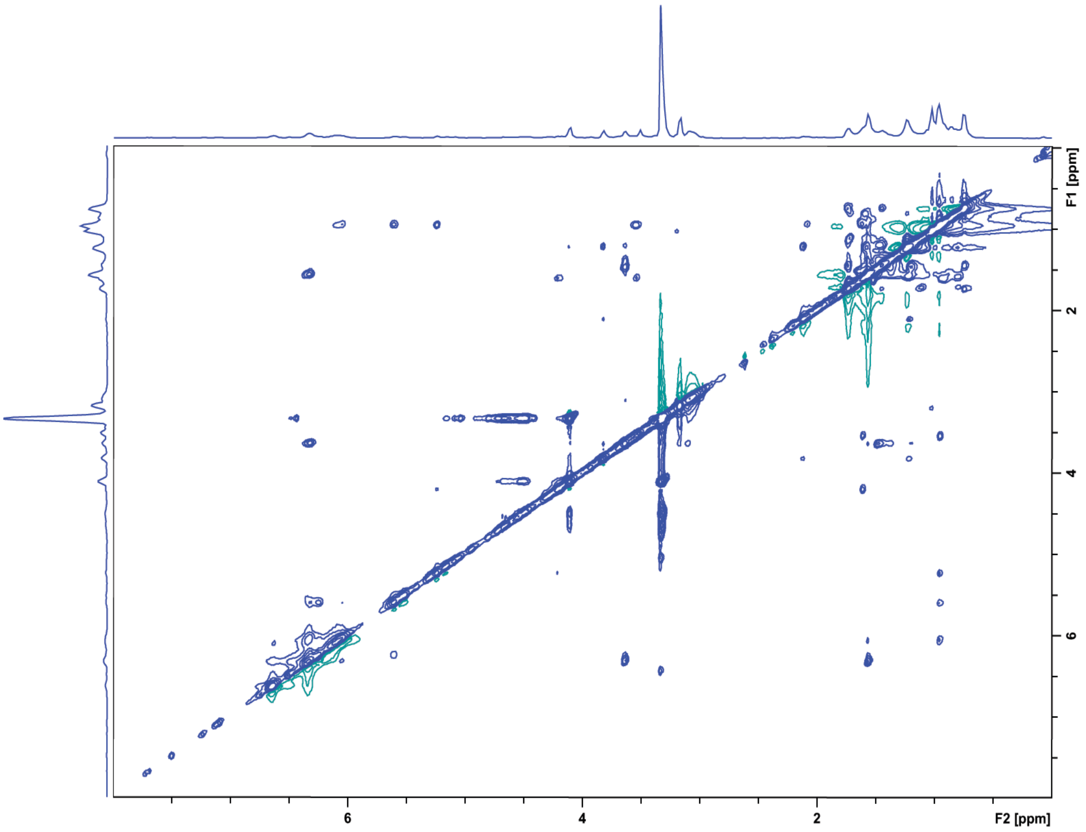

Figure S13. H,H NOESY NMR of 16-*O*-ethyl-BC231 (**7e**) with Watergate solvent-suppression of the DMSO-d_6 signal. The spectrum was recorded with a total number of scans NS = 16 and with TD(F2) = 2048 and TD(F1) = 512.

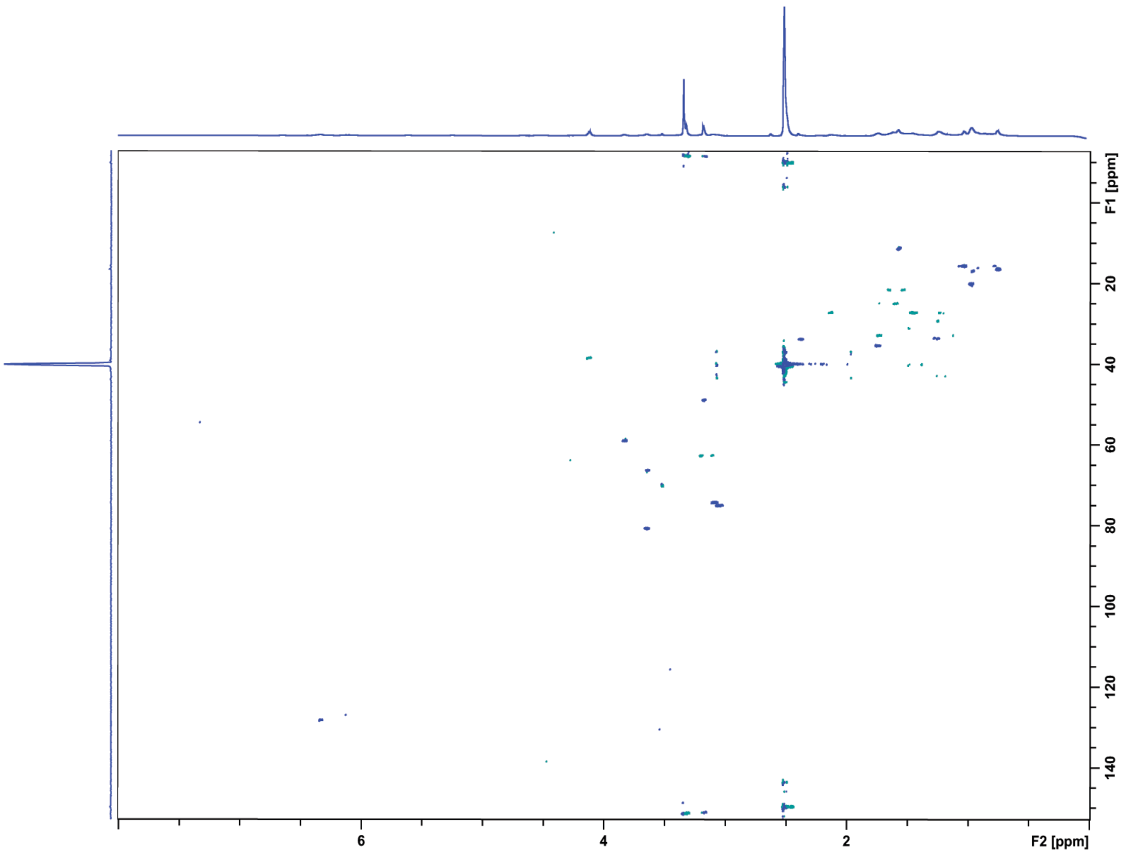

Figure S14. HSQC of 16-*O*-ethyl-BC231 **(7e).** The spectrum was recorded with a total number of scans NS = 56 and with TD(F2) = 4096 and TD(F1) = 512.

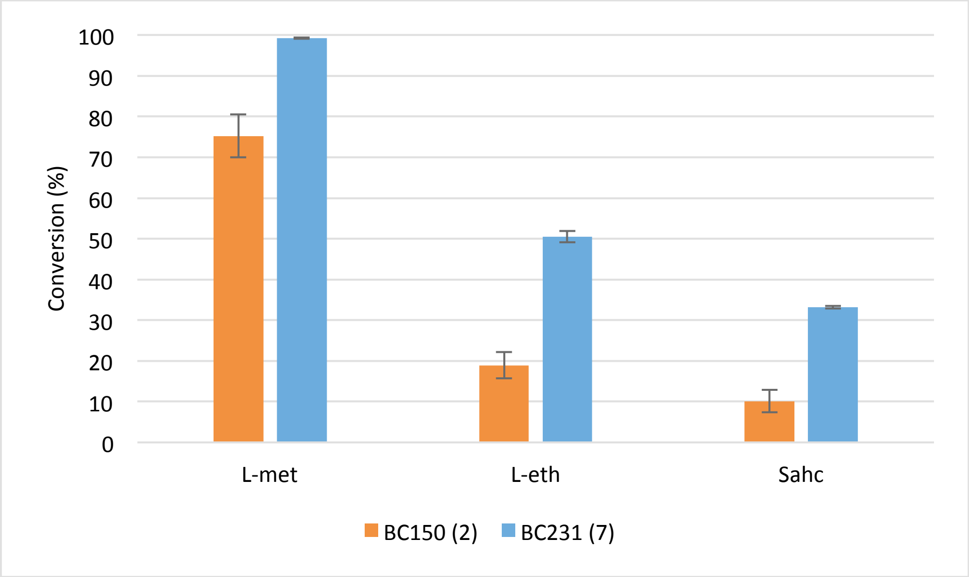

Figure S15. Relative activities of RapM with BC150 **2** and BC231 **7**. Tandem assays of RapM/hMAT2A (I322V) with L-methionine, L-ethionine and *S*-allyl-L-homocysteine were carried out with either **2** or **7** as the substrate for RapM. The assays were quenched after 60 min and the activities of RapM with both substrates compared.

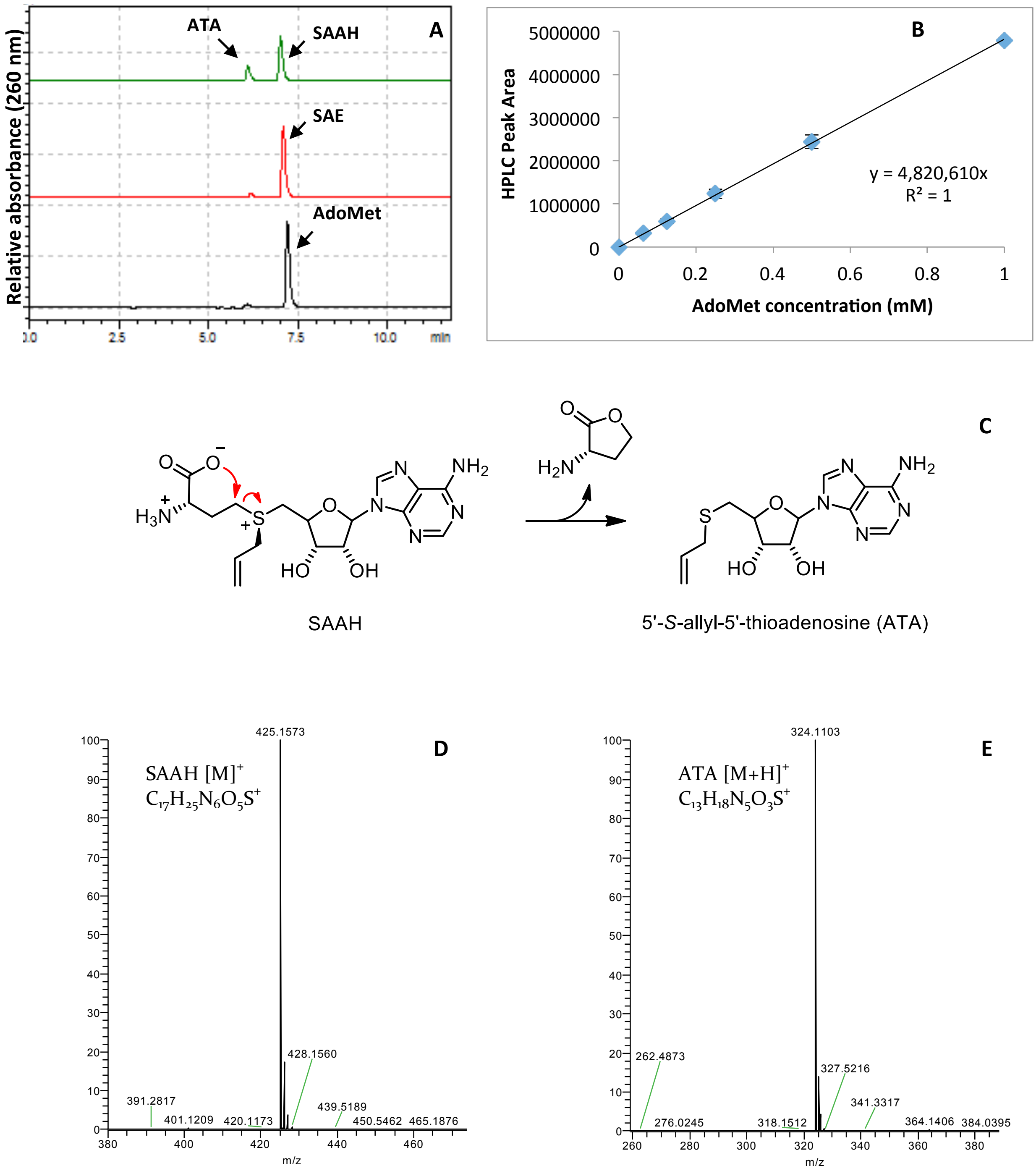

Figure S16. Enzymatic preparation of AdoMet, SAE and SAAH. **A:** AdoMet, SAE and SAAH were produced by hMAT2A (I322V), isolated using a centricon to remove the enzyme and analysed by HILIC HPLC. Retention times are: AdoMet (7.21 min); SAE (7.09 min); SAAH (7.02 min); 5′-*S*-allyl-5′-thioadenosine (ATA) (6.10 min). The SAAH sample clearly shows significant formation of the degradation product ATA (indicated by arrow) compared with the AdoMet and SAE samples; **B:** Calibration curve for AdoMet used to quantify AdoMet analogues. Injections of known concentrations of AdoMet (0, 62.5, 125, 250, 500, 1000 µM) were used to generate the calibration; **C:** The mechanism for the degradation of SAAH to ATA.[29,42,43] SAAH was incubated at 37 °C for 60 min and analysed by LC-HRMS: **D:** HRMS of SAAH showing *m*/*z* 425.1573 (calculated *m*/*z* 425.1602); **E:** HRMS of the degradation product ATA showing *m*/*z* 324.1102 (calculated *m*/*z* 324.1125).

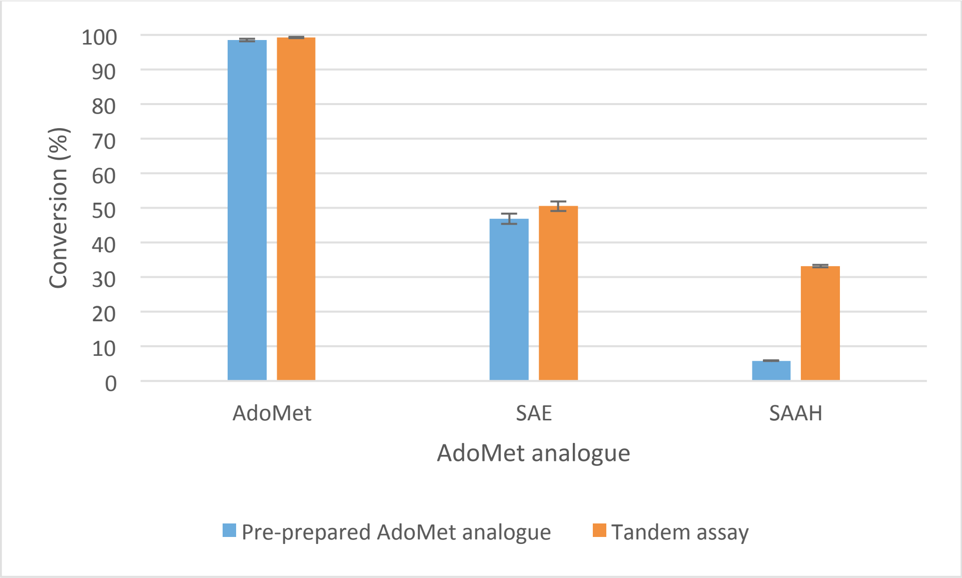

Figure S17. Activity of RapM with isolated AdoMet analogues. AdoMet, SAE and SAAH were generated enzymatically by hMAT2A (I322V), purified and quantified. Equal concentrations of each analogue were incubated with RapM and BC231 **7**. Conversion to **7m**, **7e** and **7a** reached 98%, 47% and 6% respectively, whereas in the tandem assays conversion reached 99%, 51% and 33%. The low conversion with pre-prepared SAAH suggests that SAAH or its degradation product 5′-*S*-allyl-5′-thioadenosine (ATA) may be inhibitory of RapM.

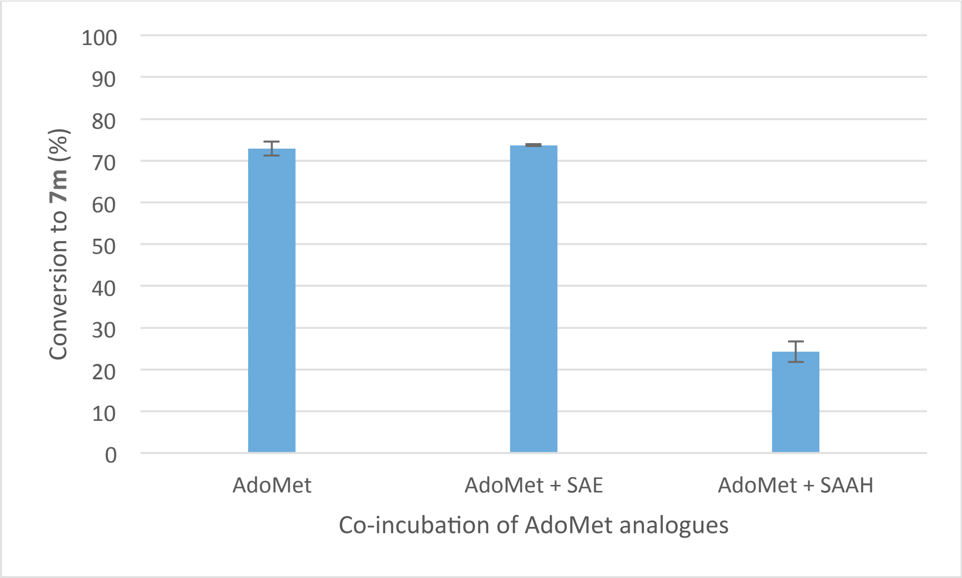

Figure S18. Effect of SAE and SAAH on RapM methylation of BC231 **7**. Incubation of RapM with BC231 **7** and either AdoMet (125 μM), AdoMet + SAE (125 μM each) or AdoMet + SAAH (125 μM each) show that SAAH is inhibitory of the methylation reaction, whereas SAE is not. No ethylated or allylated products were observed within the time period of the competitive assays (60 min).

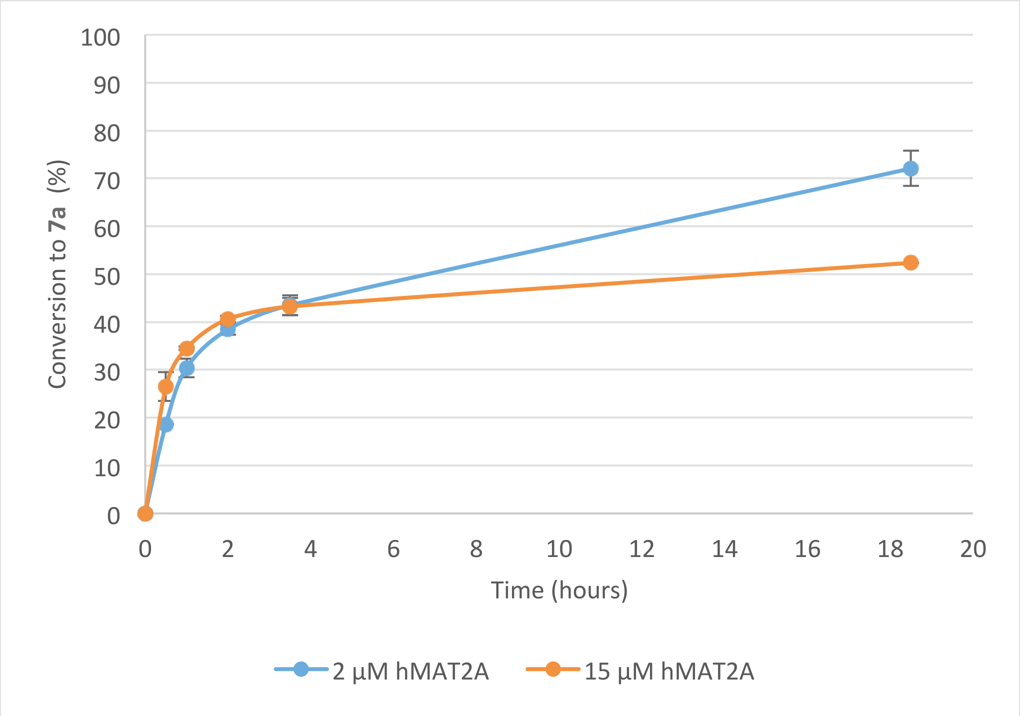

Figure S19. Effect of reduced hMAT2A concentration on rapalog allylation. The tandem assays of RapM (15 µM) with hMAT2A (I322V) (2 µM, 15 µM), and BC231 **7**, *S*-allyl-L-homocysteine and ATP, show that slower formation of the unstable SAAH co-factor results in higher overall allylated product.

Table S5. Primers used in this study.

**Primer**	**Sequence 5′-3′**	**bp**
His6-rapM F	AAAAAACATATGATGATCCAACCCGACGTCGTG	33
His6-rapM R	AAAAAAAAGCTTTTGTCTCCGTTCACACGCGGAC	34
hMAT2A I117KYT	ACAGTCACCAGATKYTGCTCAAGGTGTTC	29
hMAT2A V121KYT	TGCTCAAGGTKYTCATCTTGACAGAAATG	29
hMAT2A I322KYT	GTCTCTTATGCTKYTGGAGTTTCTCATCC	29

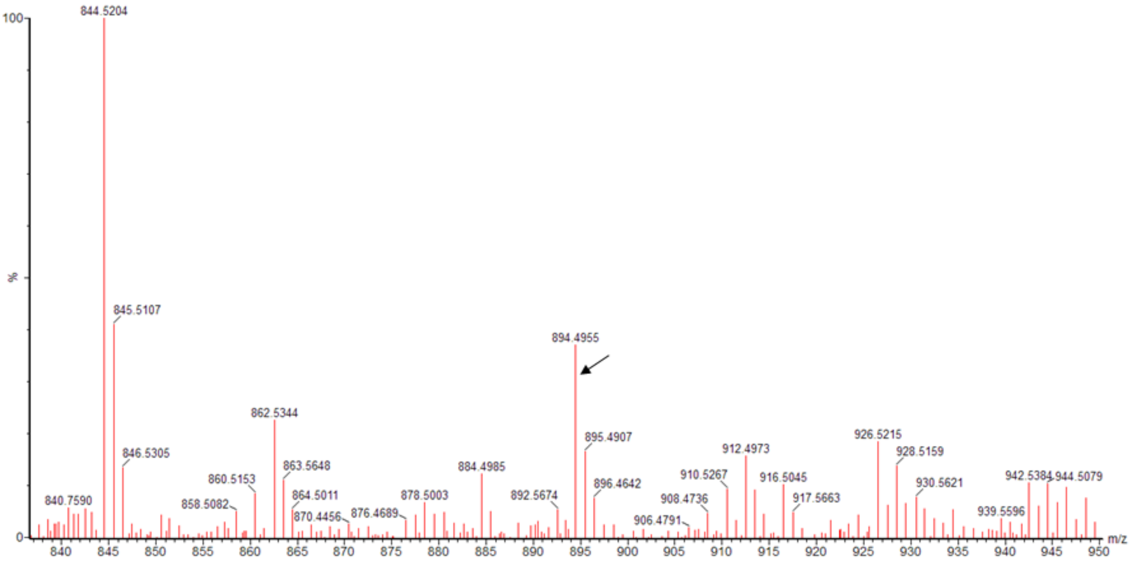

Figure S20. HRMS (ES+) of BC231 (**7**) *m/z* $[M+Na]^+$ = 894.4955 (calculated: 894.4974).

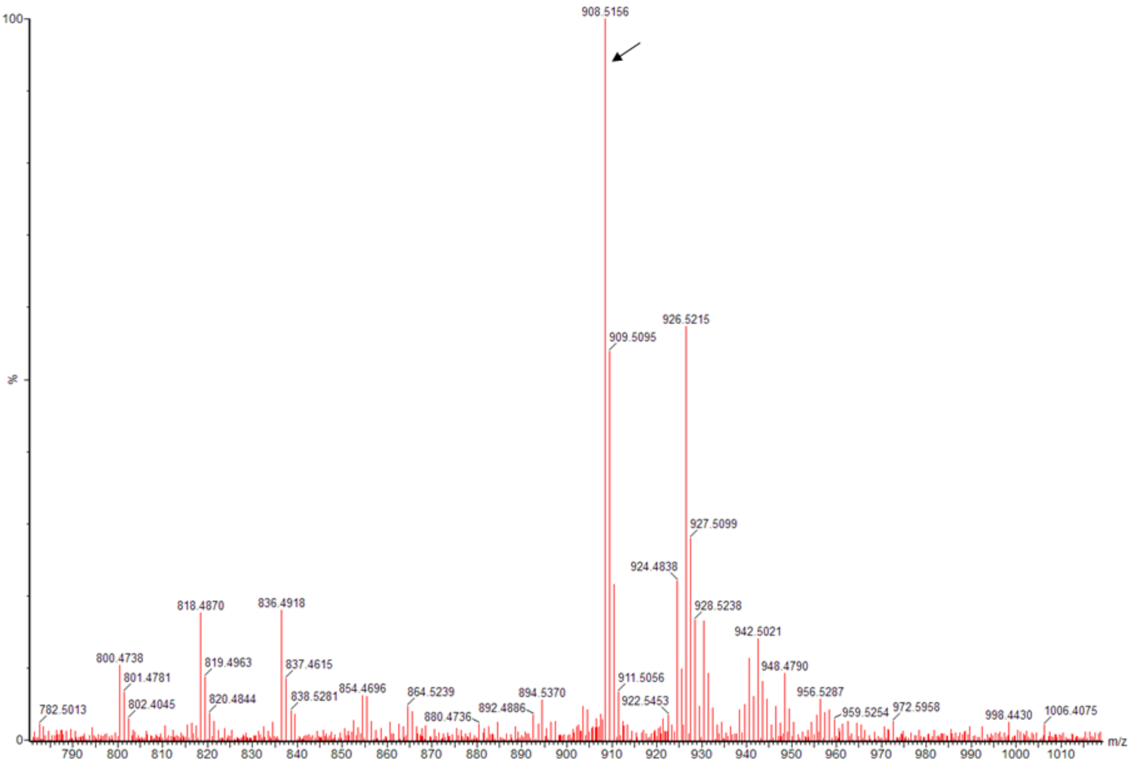

Figure S21. HRMS (ES+) of 16-*O*-methyl-BC231 (**7m**) *m/z* $[M+Na]^+$ = 908.5156 (calculated: 908.5131).

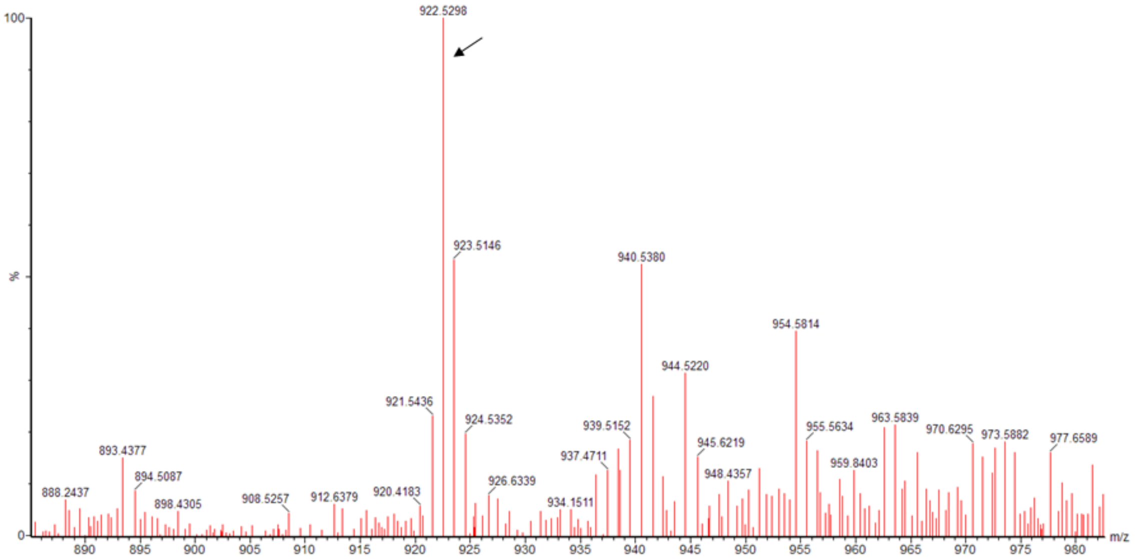

Figure S22. HRMS (ES+) of 16-*O*-ethyl-BC231 (**7e**) *m*/*z* $[M+Na]^+$ = 922.5298 (calculated: 922.5250).

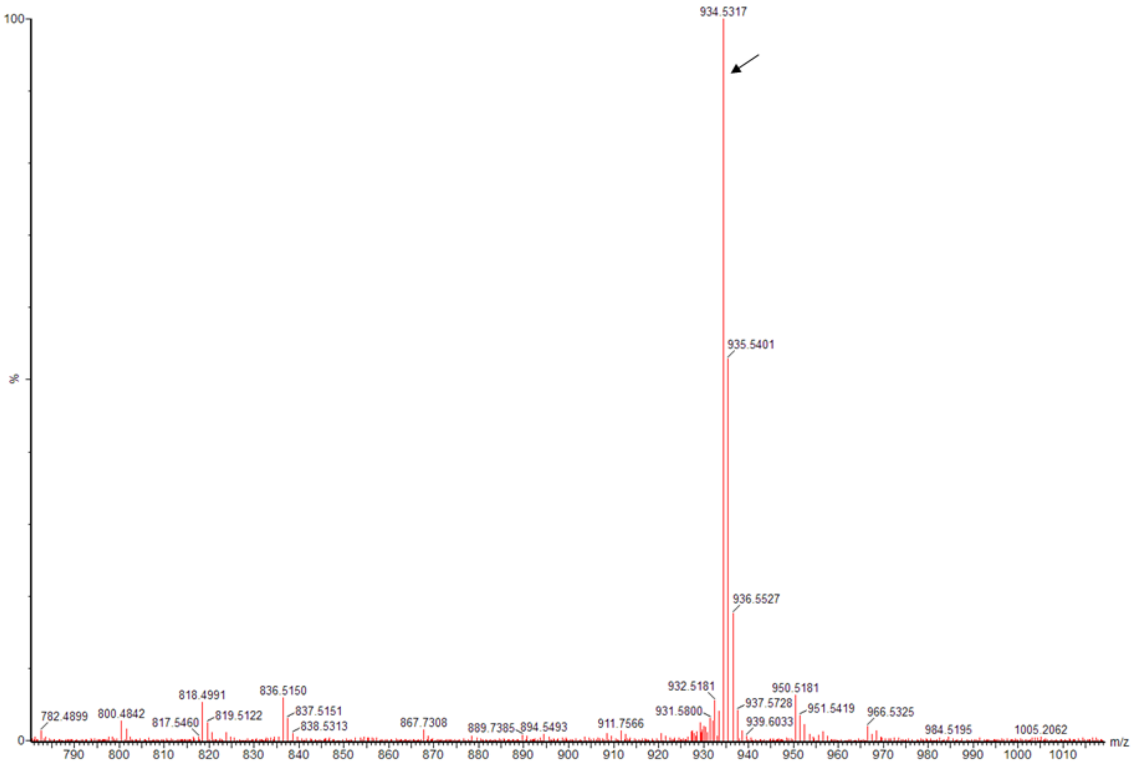

Figure S23. HRMS (ES+) of 16-*O*-allyl-BC231 (**7a**) *m*/*z* $[M+Na]^+$ = 934.5317 (calculated: 934.5287).

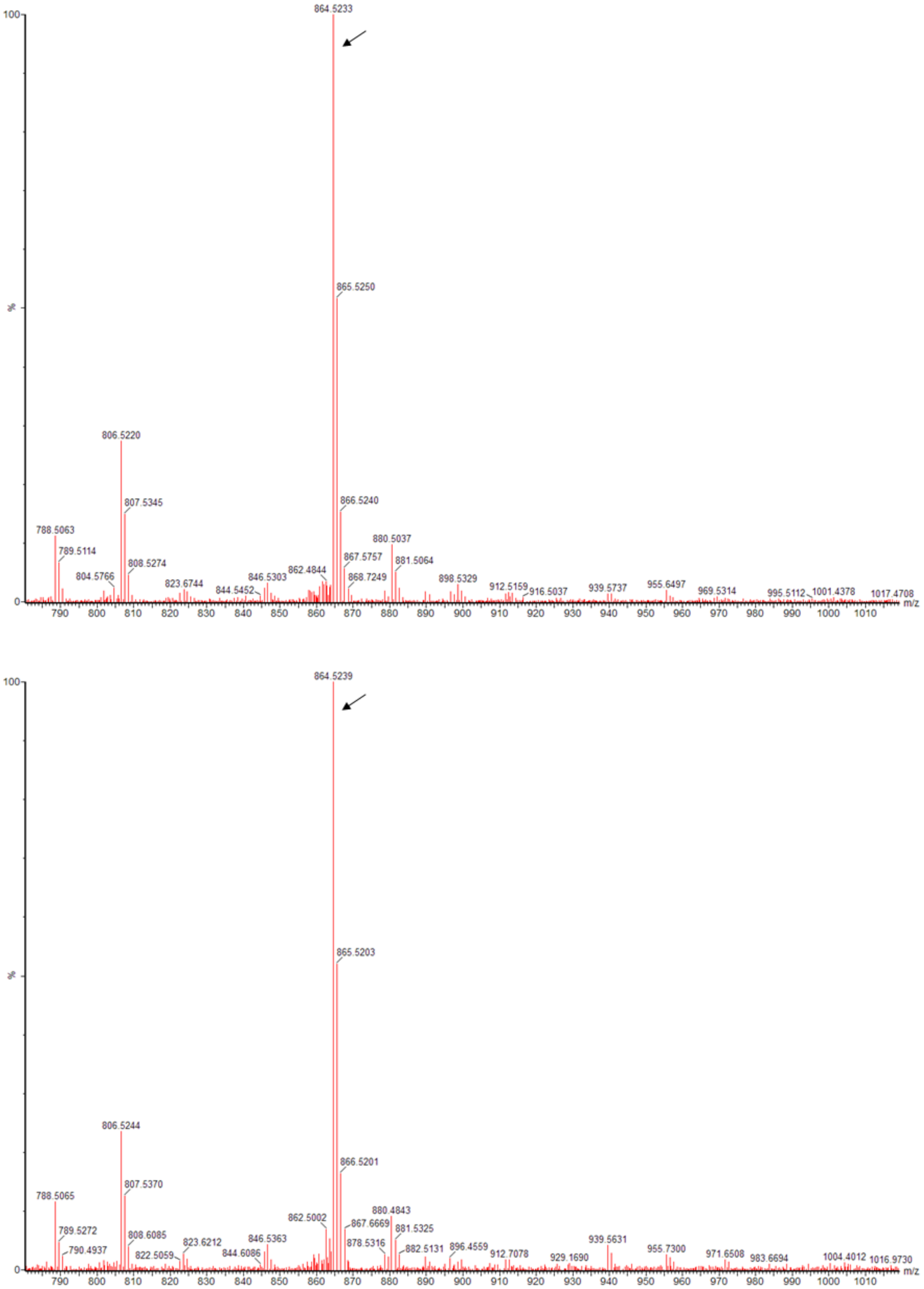

Figure S24. HRMS (ES+) of BC150 (**2**) *m*/*z* $[M+Na]^+$ = 864.5233, 864.5239 (calculated: 864.5232).

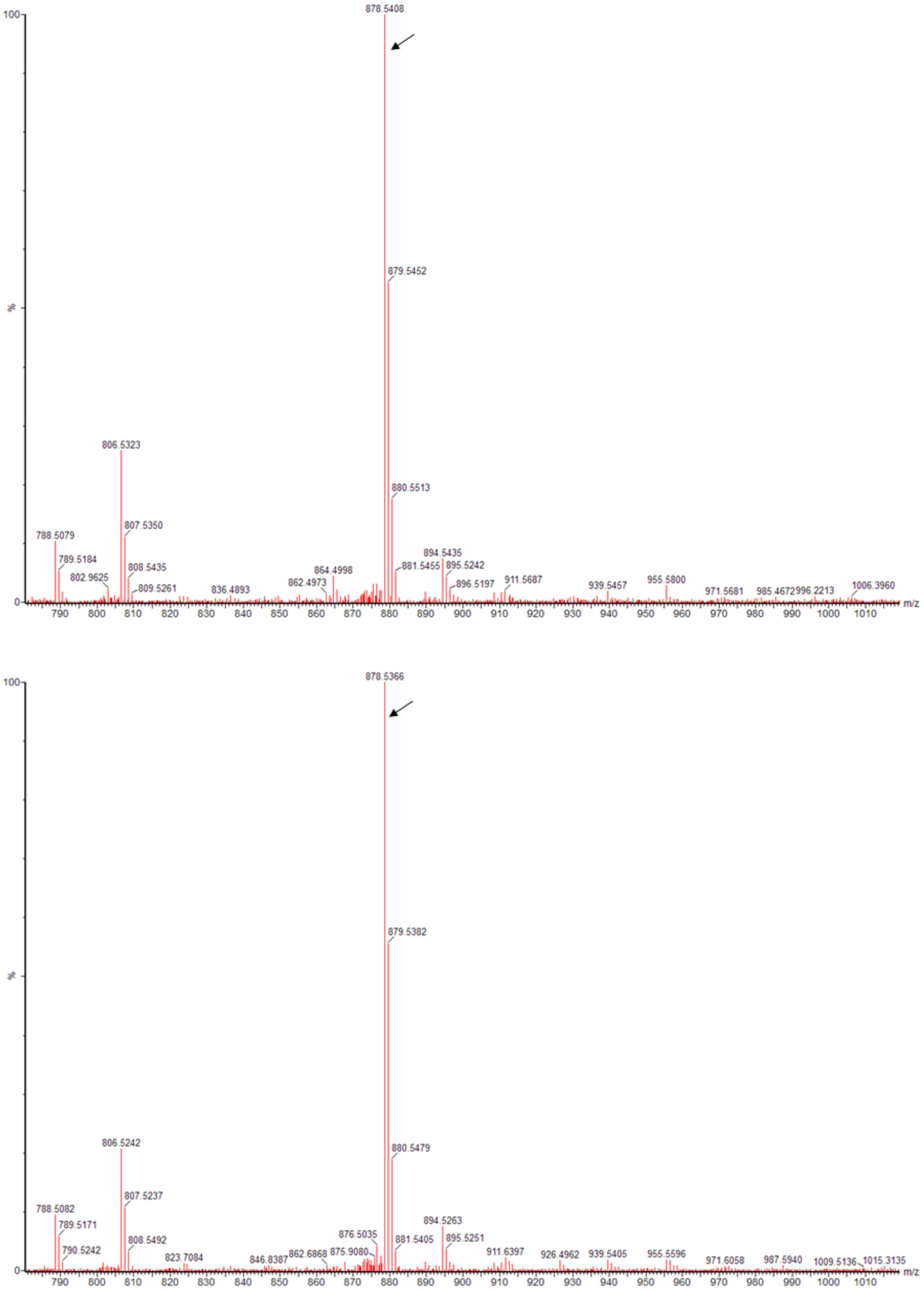

Figure S25. HRMS (ES+) of 16-*O*-methyl-BC150 (**2m**) *m/z* $[M+Na]^+$ = 878.5408, 878.5366 (calculated: 878.5389).

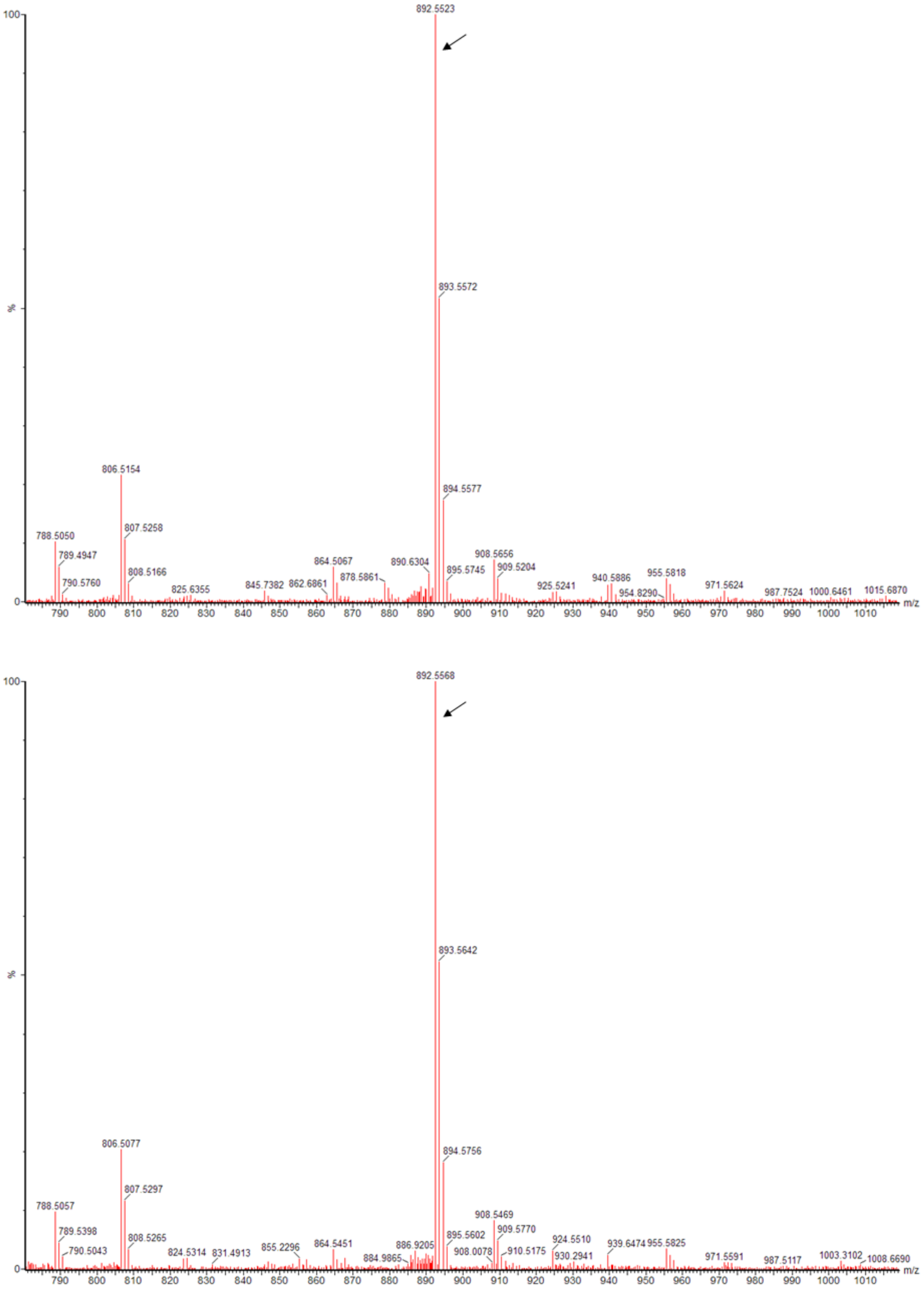

Figure S26. HRMS (ES+) of 16-*O*-ethyl-BC150 (**2e**) *m/z* $[M+Na]^+$ = 892.5523, 892.5568 (calculated: 892.5545).

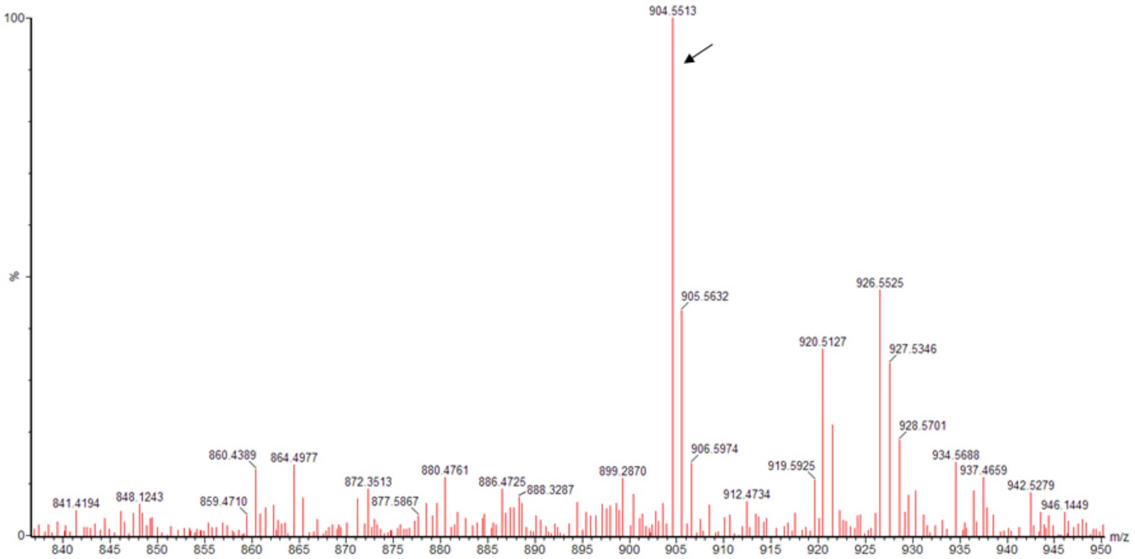

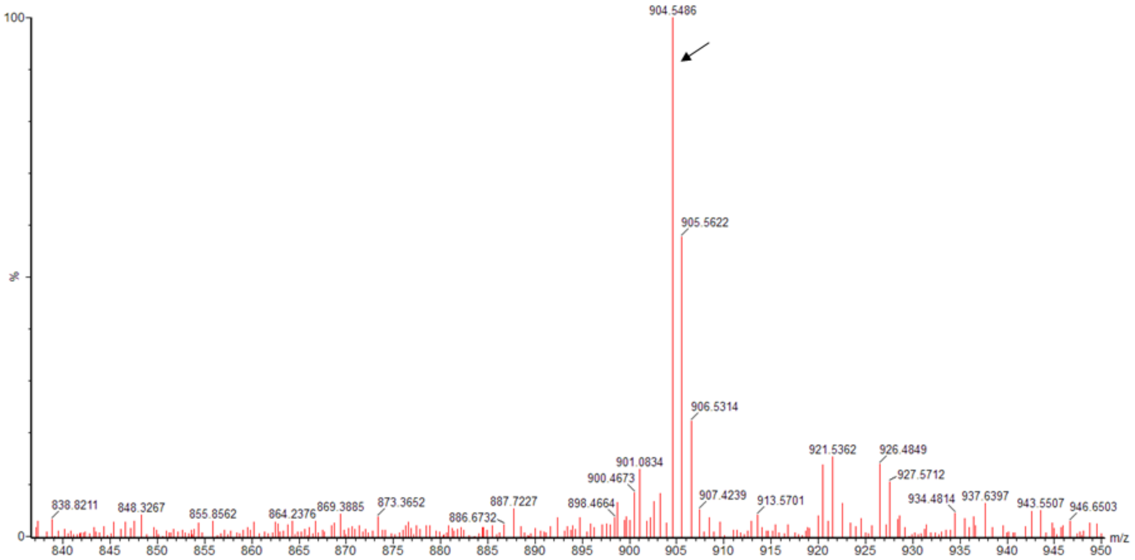

Figure S27. HRMS (ES+) of 16-*O*-allyl-BC150 (**2a**) *m/z* $[M+Na]^+$ = 904.5513, 904. 5486 (calculated: 904.5545).
